# Modulation of Neuronal Potassium Channels During Auditory Processing

**DOI:** 10.3389/fnins.2021.596478

**Published:** 2021-02-03

**Authors:** Jing Wu, Leonard K. Kaczmarek

**Affiliations:** Department of Pharmacology, Yale University School of Medicine, New Haven, CT, United States

**Keywords:** cocktail party effect, sound localization, MNTB, potassium channels, firing pattern

## Abstract

The extraction and localization of an auditory stimulus of interest from among multiple other sounds, as in the ‘cocktail-party’ situation, requires neurons in auditory brainstem nuclei to encode the timing, frequency, and intensity of sounds with high fidelity, and to compare inputs coming from the two cochleae. Accurate localization of sounds requires certain neurons to fire at high rates with high temporal accuracy, a process that depends heavily on their intrinsic electrical properties. Studies have shown that the membrane properties of auditory brainstem neurons, particularly their potassium currents, are not fixed but are modulated in response to changes in the auditory environment. Here, we review work focusing on how such modulation of potassium channels is critical to shaping the firing pattern and accuracy of these neurons. We describe how insights into the role of specific channels have come from human gene mutations that impair localization of sounds in space. We also review how short-term and long-term modulation of these channels maximizes the extraction of auditory information, and how errors in the regulation of these channels contribute to deficits in decoding complex auditory information.

## Introduction

**FIGURE 1:**
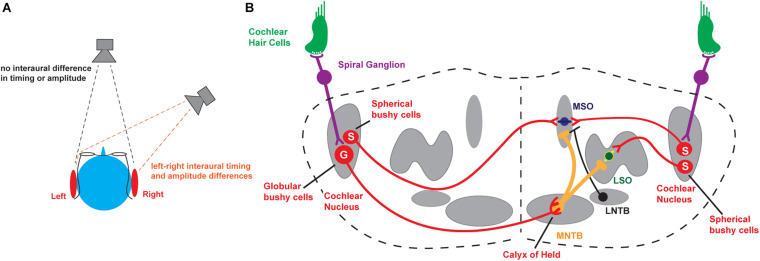
Binaural hearing. **(A)** Schematic illustrating interaural comparisons. **(B)** Brainstem circuits underlying the detection of interaural timing and intensity differences. MNTB, medial nucleus of the trapezoid body; MSO, medial superior olive; LSO, lateral superior olive; S, Spherical bushy cells; G, Globular bushy cells. For completeness, the lateral nucleus of the trapezoid body (LNTB), which sends inhibitory input to the MSO, is also depicted.

The ability to discriminate and isolate a particular source of sound in a complex auditory environment, also referred to as the *cocktail party effect*, is a remarkable feature of the human auditory system (Haykin and Chen, 2005; McDermott, 2009). It requires neurons in auditory brainstem nuclei to encode aspects of a sound, such as its timing, frequency, and intensity, and then to compare differences in these characteristics in the inputs coming from the two ears. A major cue that is used to discriminate the location of a sound in space is its time of arrival at the two ears. A sound that arrives at the right ear earlier than at the left will be perceived as coming from the right, while one that arrives at both ears simultaneously will appear to originate in front of (or directly behind) the listener (Figure 1A). Similarly, a higher intensity of sound at the right ear will promote the impression that the sound originates on the right. Other cues, such as the frequency distribution within the sound, contribute to detection of sound location, particularly in distinguishing sounds coming from above, below or behind the listener (Tollin, 2009; Grothe et al., 2010; Grothe and Pecka, 2014; Yin et al., 2019). Nevertheless, the time of arrival appears to be an essential cue in distinguishing sound location and is essential to a person’s ability to focus attention to content originating in one specific location and to ignore multitudes of sounds originating elsewhere (i.e., the cocktail party effect). Humans can readily detect interaural time differences of tens of microseconds, much less than the duration of a neuronal action potential (Carr and MacLeod, 2010).

The major neuronal circuits that detect interaural differences in timing and intensity of sounds are located in the brainstem and are illustrated in Figure 1B. Along with other aspects of an auditory stimulus, the information that is used for the localization of sounds is first detected and transduced by auditory hair cells and then transmitted through neurons in the spiral ganglion to bushy cells in the anteroventral cochlear nucleus (AVCN), faithfully preserving information about the frequency, intensity, and timing of each stimulus. In turn, spherical bushy cells relay this information to the medial and lateral superior olivary nuclei (MSO and LSO) where interaural time and intensity differences from the two cochleae are computed and compared directly (Tollin, 2003, 2009; Grothe et al., 2010; Grothe and Pecka, 2014; Yin et al., 2019). An intermediate nucleus of this circuit is the medial nucleus of the trapezoid body (MNTB). The MNTB is an inverting relay, receiving excitatory inputs from the contralateral globular bushy cells and providing ipsilateral glycinergic inhibition to both the MSO and LSO (Moore and Caspary, 1983). The firing pattern of each MNTB neuron is dominated by an excitatory synaptic input from a giant presynaptic terminal, called the *calyx of Held*, located at the end of the axons of the globular bushy cells of the AVCN (Held, 1893; Banks and Smith, 1992; Forsythe and Barnes-Davies, 1993). The calyx of Held synapse targets most of the cell body, providing very secure and accurately timed excitation of the MNTB neurons that *in vivo* may enable spatial localization of sound transients (Joris and Trussell, 2018). The large calyx of Held has been used widely to investigate presynaptic ion channels, neurotransmitter release, and synaptic plasticity (for reviews see Schneggenburger and Forsythe, 2006; Borst and Soria van Hoeve, 2012; Baydyuk et al., 2016). Many of the studies of how ion channels become modified by stimulation in the auditory brainstem have been carried out using the principal neurons of the MNTB, as well as these specialized presynaptic terminals.

## Potassium Channels in Auditory Brainstem Neurons

Most of the neurons in pathways that provide inputs to the MSO and LSO have two critical features that distinguish them from most other neurons in the nervous system. First, they are capable of firing at very high rates of up to 800 action potentials/second or more (Taschenberger and von Gersdorff, 2000; Kopp-Scheinpflug et al., 2003b). Second, they lock their action potentials with microsecond precision to the specific phase of a sound with a frequency of <2 kHz or so. Neurons with characteristic frequencies >2 kHz lock their action potentials to the envelope of amplitude-modulated high frequency sounds. These high frequency neurons are also typically able to phase lock to lower frequencies even better than neurons with lower characteristic frequencies (Joris et al., 1994).

**FIGURE 2:**
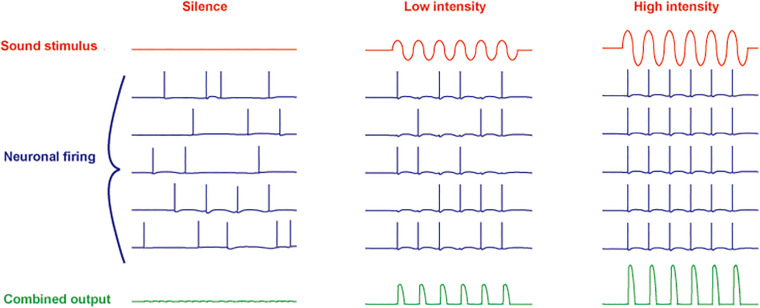
Schematic diagram of responses of five neurons to increasing intensity of a sinusoidal sound stimulus. A combined ensemble average of the output is illustrated in the bottom traces. In silence, spontaneous activity (originating in spontaneous release of neurotransmitter from cochlear hair cells) in all neurons is uncorrelated (*left*). At low sound intensities, the action potentials become entrained to the stimulus with no overall change in firing rate (*center*). At high intensity, the firing rate of the neurons is increased with all action potentials phase-locked to the stimulus (*right*).

Another feature of neurons such as those in the MNTB is their ability to extract auditory information even with little or no change in overall firing rate. Even in silence, MNTB neurons are driven to fire at rates from 10 to ∼ 200 Hz by afferent activity that originates as spontaneous release of transmitter from hair cells (Brownell, 1975; Hermann et al., 2007; Kopp-Scheinpflug et al., 2008). At low intensities of sound stimulation, however, the action potentials become entrained to the auditory stimulus (Figure 2). As the intensity of sounds is increased, the firing rate of MNTB neurons can be pushed to over 800 Hz. Thus, these neurons respond to changes in both the timing and intensity of auditory stimuli.

**FIGURE 3:**
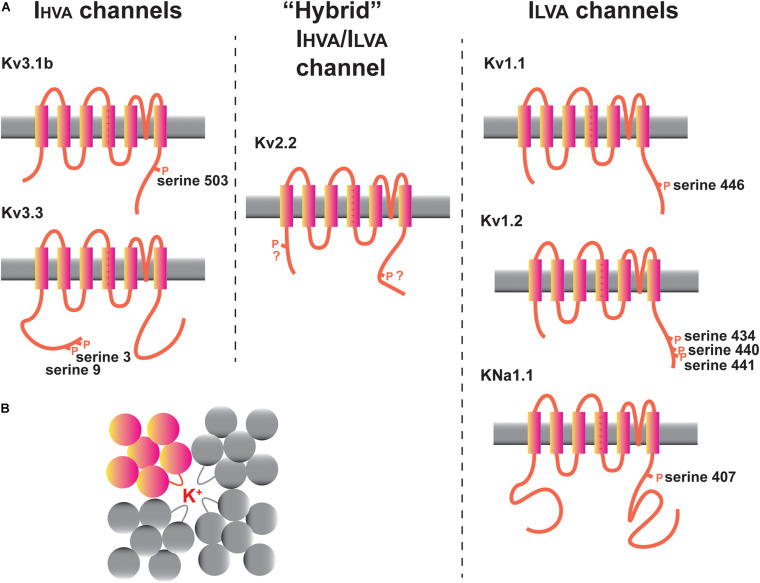
Potassium channels in the MNTB. **(A)** Diagrams illustrating transmembrane topology and some of the regulatory phosphorylation sites in I_HVA_ (Kv3 family) and I_LVA_ (Kv1 and K_Na_ families) channels with documented functions in the MNTB. The intermediate I_HVA/_I_LVA_ Kv2 family Kv2.2 channel is also depicted. **(B)** “Top down” schematic diagram illustrating the assembly of four Kv family subunits into a tetrameric channel with the potassium ion conduction pathway in the center.

These ability of auditory brainstem neurons to transmit auditory information at these high rates requires them to have membrane properties appropriate for rapid transduction of synaptic inputs into outgoing trains of action potentials. The proteins that determine the intrinsic excitability of neurons are ion channel proteins and the auxiliary proteins and enzymes that directly determine their biophysical properties. The major pore-forming α-subunits that constitute the core of each ion channel are selective for sodium, calcium, potassium or chloride ions, and each of these is critical to defining the way a neuron responds to stimulation (Leao, 2019; Rasmussen and Trimmer, 2019; Kaczmarek, 2020). Among these pore-forming subunits, the most diverse group is that of the α-subunits for channels selective for potassium ions. The number of known genes that encode potassium channel α-subunits (77 genes) is greater than that for all the other subunits combined (Kaczmarek, 2020). Moreover, in most cases, the pore of a potassium channel is formed by a tetramer of α-subunits, which can be the product of the same or a different gene (Figures 3A,B). Alternative splicing of the mRNAs for most of the α-subunits, coupled with the fact that each subunit can be modified by posttranslational events such as phosphorylation or assembly with auxiliary subunits, provides a near infinite number of possibilities to regulate properties of potassium channels.

Potassium channels can be categorized into five groups, each of which has different gating and pharmacological properties and contributes in a different way to aspects of excitability (Alexander et al., 2019). These are (1) voltage-dependent channels of the Kv family, (2) calcium-activated channels (K_Ca_ channels), (3) sodium-activated channels (K_Na_ channels), (4) inwardly rectifying channels (K_ir_ channels), and (5) two pore domain channels (K_2P_ channels). Recent work has also indicated that some of these channels regulate other aspects of cell biology beyond membrane excitability, so called non-conducting functions (Kaczmarek, 2006; Lee et al., 2014; Rasmussen and Trimmer, 2019). The channels that have received the most amount of experimental attention in auditory brainstem neurons, particularly in the MNTB, are depicted in Figure 3A. These are the Kv1.1, Kv1.2, Kv2.2, Kv3.1, and Kv3.3 channel subunits of the Kv family and the sodium dependent K_Na_ channels. Several other channels, whose functions are less well understood, including Kv1.6, Kv3.4, Kv4.3, Kv11 channels, and the two-pore domain subunits K_2P_1 and K_2P_15 will also be discussed.

## Human Mutations That Impact Sound Localization

Some key insights into which potassium channels play key roles in auditory neurons have come from the study of neurological disorders that are associated with deficits in processing auditory information, particularly the localization of sounds in space. These include autism, Fragile X syndrome, and certain ataxias (Middlebrooks et al., 2013; Ferron, 2016; McCullagh et al., 2020). Fragile X syndrome, the leading known cause of inherited intellectual disability, results from mutations that suppress the expression of Fragile X Mental Retardation Protein (FMRP), a mRNA binding protein that controls the function and the expression level of a variety of proteins including several ion channels such as the Kv3.1, Kv3.3, Kv1.2 and K_Na_1.1channels (Darnell et al., 2001; Brown et al., 2010; Strumbos et al., 2010a; Zhang et al., 2012; Yang et al., 2018). The characteristics of these channels will be described later. Fragile X patients are hypersensitive to auditory stimuli (St Clair et al., 1987; Arinami et al., 1988; Rojas et al., 2001; Castren et al., 2003; Roberts et al., 2005; Van der Molen et al., 2012) and are impaired in their ability to discriminate interaural timing, rendering them unable to localize sounds (Hall et al., 2009; Rotschafer and Razak, 2014). A second genetic condition is Spinocerebellar Ataxia type 13 (SCA13), a movement disorder caused by mutations in the gene encoding the Kv3.3 channel (Zhang and Kaczmarek, 2016). These mutations cause either early-onset or late-onset motor disabilities and different mutations result in either a loss of Kv3.3 current or a change in its voltage-dependence or kinetics. Nevertheless, even in the case of the late-onset disease, younger SCA13 patients with no motor symptoms are unable to discriminate interaural timing differences of up to a millisecond (Middlebrooks et al., 2013).

## Voltage-Dependence and Kinetics of a Potassium Channel Determines Its Effect on Excitability: I_HVA_ and I_LVA_ Channels

**FIGURE 4:**
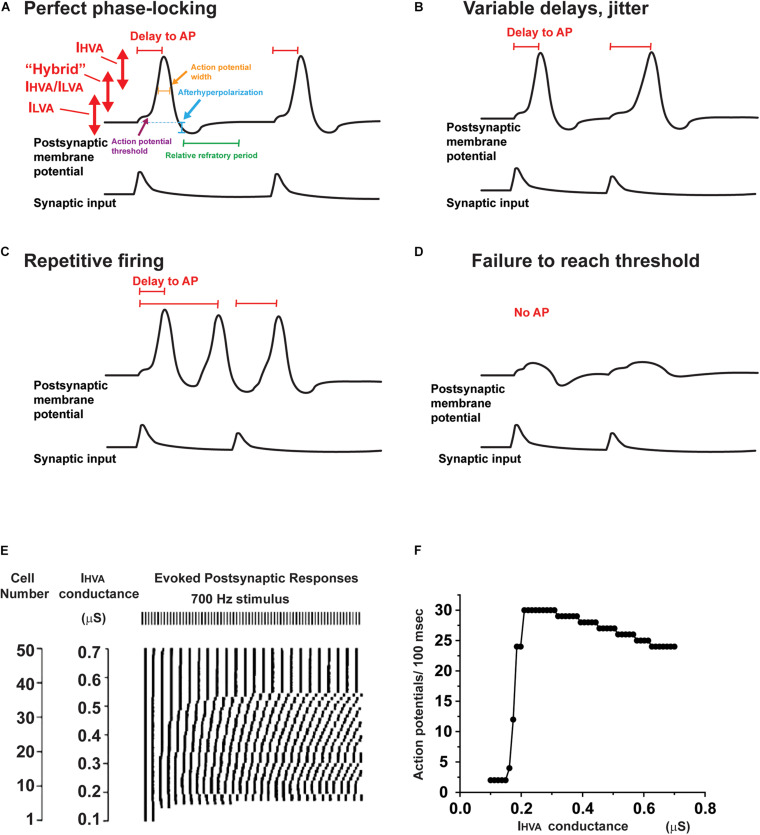
Effects of altering potassium conductances on firing patterns. **(A)** Illustration of the voltage ranges in which I_HVA_ and I_LVA_, as well as intermediate potassium channels operate during the response of a neuron to two consecutive synaptic inputs. Changes in these potassium currents are able to alter the resting potential, action potential threshold, height and width, as well as the afterhyperpolarization and refractory period that follows each action potential. **(B–D)** A change in potassium currents alter the response of a neuron from a response like that in panel **(A)** to one with variable delays in response to each synaptic input **(B)**, repetitive firing that is not locked to the synaptic input **(C)** or failure to evoke an action potential **(D)**. **(E)** Results of numerical simulations showing that the timing of action potentials evoked by a regular 700 Hz stimulus differs in neurons that have different levels of I_HVA_ currents. The timing of the evoked action potentials is presented in raster plot under the stimulus pulses (Kaczmarek, 2012). **(F)** A plot of firing rate as a function of the different levels of I_HVA_ currents in panel **(E)**. Note that, although the firing rate changes by only a small amount over wide ranges of I_HVA_, the timing of the action potentials with respect to the stimuli (shown in panel **E**) is very sensitive to small changes in I_HVA_.

**FIGURE 5:**
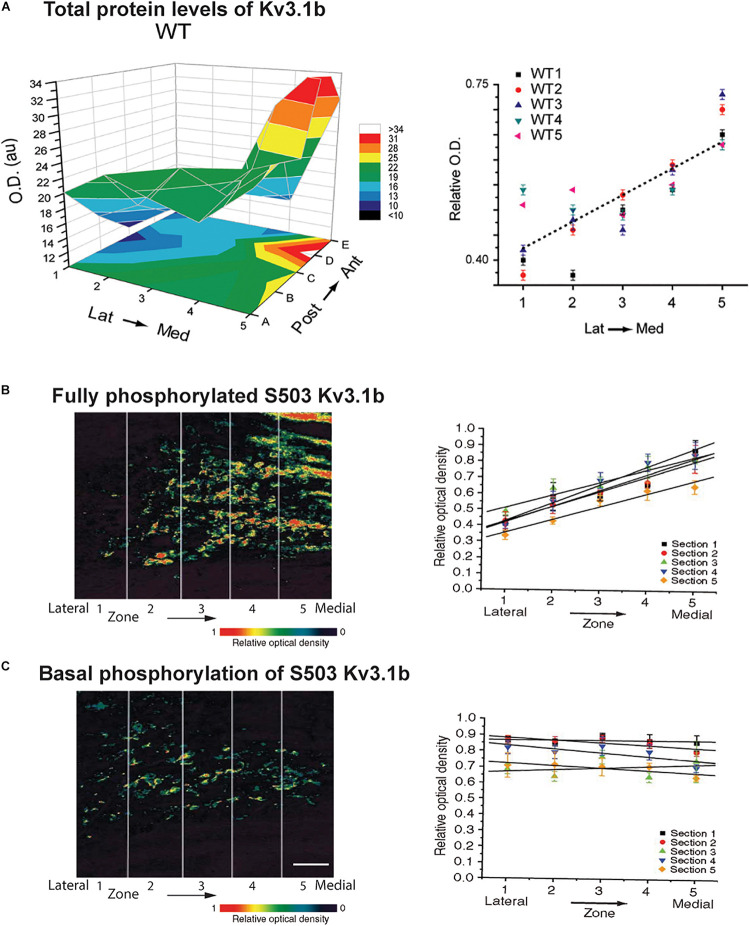
Tonotopic gradients of Kv3.1b and phosphorylated Kv3.1b in the MNTB. **(A)** Representative three-dimensional plot of average Kv3.1b immunoreactivity (OD) in each of 25 stereotaxic zones along the lateral to medial and posterior to anterior axes in a mouse MNTB. Graph at right plots relative Kv3.1b immunoreactivity across the medial-lateral axis for five different animals (Strumbos et al., 2010a). **(B,C)** Tonotopic gradients of Kv3.1b phosphorylation in MNTB treated with a PKC activator to maximally stimulate phosphorylation of the channel **(B)** and in resting unstimulated MNTB slices **(C)**. Left panels show pseudocolor images of serine 503 phosphorylation of Kv3.1b detected with a phospho-specific antibody. Right panels show quantification of phosphorylation in five tonotopic zones along the lateral to medial axis (*n* = 5 sections in each case). A clear tonotopic gradient, matching that of total Kv3.1b protein is seen when channels are all maximally phosphorylated **(B)**. No gradient is, however, detected under resting conditions, indicating that the degree of phosphorylation is reduced in the medial high frequency MNTB neurons (Song et al., 2005). Scale bar, 200 μm.

**FIGURE 6:**
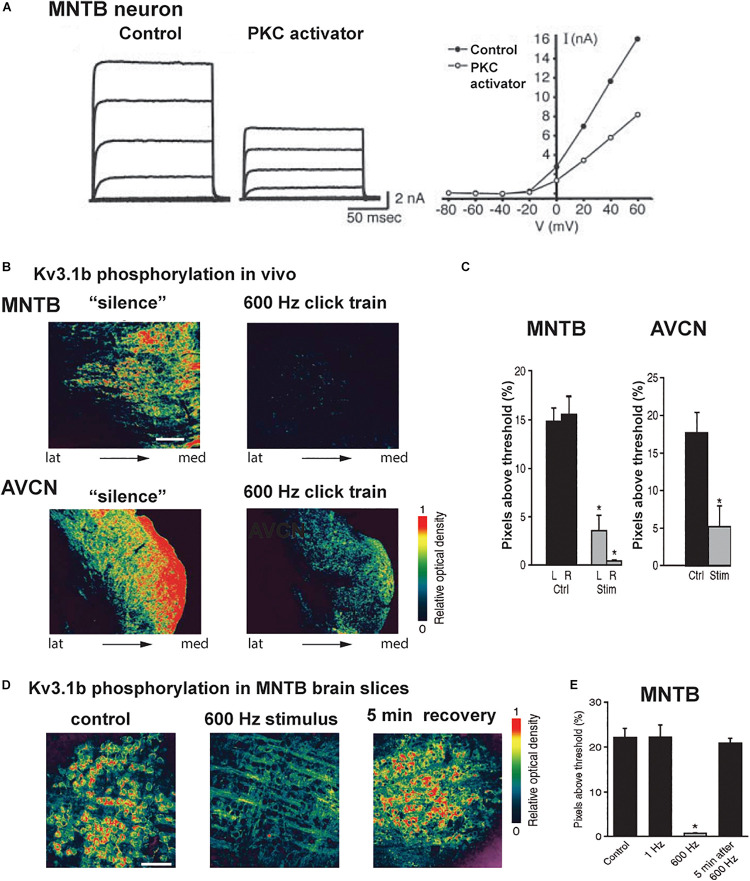
Kv3.1b phosphorylation is reduced *in vivo* and *in vitro* after auditory or synaptic stimulation. **(A)** Voltage clamp traces and current-voltage relations of isolated I_HVA_ current in an MNTB neuron showing that current is reduced after exposure to an activator of PKC (Macica et al., 2003). **(B)** Pseudocolor images of phospho-Kv3.1b immunostaining in an MNTB (*top*) and AVCN (*bottom*) from animals kept in a soundproof room or exposed to stimulation with a 600-Hz click train (70 dB SPL, sound pressure level for 5 min). **(C)** Quantification of the phospho-Kv3.1 immunofluorescence in MNTB and AVCN (Ctrl, no stimulation; Stim, 600 Hz click train). **(D)** Pseudocolor images of phospho-Kv3.1b immunostaining in MNTB principal neurons in brain slices, under control conditions (*left*) and after stimulation at 600 Hz for 20 s (*center*) and 5 min after 600-Hz stimulation (*right*). Scale bar, 100 μm. **(E)** Quantification of change in phosphorylation for the three conditions in panel **(C)**, as well as for a low rate of stimulation (1 Hz for 20 s). In panels **(C,E)**, *p < 0.05 when compared to control (Song et al., 2005).

In auditory neurons as in other excitable cells, potassium channels regulate several key aspects of intrinsic excitability (Figure 4). These aspects include: (1) the resting membrane potential, (2) the width of an action potential (Figure 4A), (3) the threshold for generation of an action potential (Figures 4A,D), (4) the timing of an action potential following a synaptic input (Figures 4A,B), (5) whether the neuron fires multiple action potentials in response to stimulation, and, if so, the frequency of firing (Figure 4C), and (6) the amount of neurotransmitter released at a synaptic ending following a presynaptic action potential.

Because the efflux of potassium ions during the opening of a channel hyperpolarizes neurons, a common perception is that increases in potassium currents reduce excitability. This is, however, not always the case. The opening of many potassium channels, particularly those in the Kv family, depends on the membrane potential. If a potassium channel opens at the resting potential, this will indeed hyperpolarize the cell and reduce excitability. A subset of channels, however, open rapidly only at very positive potentials, i.e., only during an action potential as the membrane potential approaches 0 mV. An increase in the number or activity of such channels, which speeds repolarization of action potentials without affecting threshold, actually increases neuronal excitability because it reduces the inactivation of sodium channels. The duration of action potentials (∼ 200 μ s) in many auditory brainstem neurons is less than the time constant for inactivation of sodium current in the same cells (Leao et al., 2006a; Scott et al., 2010). Thus, limiting the height and width of action potentials reduces the amount of sodium current inactivation with a single action potential. Moreover, rapid repolarization by potassium currents increases the proportion of time spent at negative potentials, accelerating recovery from inactivation. Thus, in both numerical simulations and experiments with transfected neurons, increasing the number of potassium channels that open rapidly at positive potentials markedly enhances the ability to fire at high rates (Kaczmarek et al., 2005; Song et al., 2005).

Potassium currents that activate near the resting potentials have been termed “low-voltage activated” or I_LVA_ currents, while those that activate selectively during action potentials have been termed “high-voltage activated” or I_HVA_ currents. This distinction is particularly important for auditory brainstem neurons, which fire at hundreds of Hz. Figure 3A separates some of the channels expressed in MNTB neurons into these two groups, together with one channel (Kv2.2) that falls between these two extremes. The approximate voltage range in which these classes of channel begin to activate is depicted in Figure 4A.

## Ion Channels Can Be Rapidly Modified by Second Messenger Pathways

All ion channel proteins can be modified by posttranslational mechanisms, such as protein phosphorylation. Some modifications occur after a channel has been synthesized and may influence its stability to proteases, its location and the rate at which it is trafficked or inserted into the plasma membrane (Yang et al., 2007b; Vacher and Trimmer, 2011). Other processes can rapidly modify either the current amplitude or kinetics of a channel once it has been in placed in the membrane. Figure 3A indicates some of the phosphorylation sites on the I_HVA_ and I_LVA_ channels present in the MNTB which have been demonstrated to modify the currents that flow through these channels. Clearly, modulation of potassium currents has the potential to alter the way a neuron responds to precisely timed input (Kaczmarek, 2012). For example, in Figure 4A, a single action potential is accurately locked to each synaptic depolarization. An alteration in potassium currents could change a response like that in Figure 4A to one with a variable delay from stimulation to the onset of an action potential (jitter, Figure 4B), an overabundance of evoked action potentials (Figure 4C) or failure to evoke an action potential in response to synaptic depolarization (Figure 4D).

At first, one might assume that the ability to fire at a high rate with high temporal accuracy might require an invariant set of potassium currents that cannot be modulated. However, a fixed set of potassium currents that provide optimal locking to one pattern of stimulation (e.g., that evoked by low frequency sounds at low intensity) may fail to generate an adequate response to another pattern (e.g., high frequency sounds at high intensity). This is evident in numerical simulations such as those in Figure 4E, which depicts a raster plot of the timing of action potentials evoked by a 700 Hz stimulus in 50 different neurons, which all have the same amplitude of I_LVA_ but have different levels of I_HVA_ currents (Kaczmarek, 2012). The timing of the responses varies substantially such that at low levels of I_HVA_, firing in response to the stimulus train cannot be sustained, while regular responses are evoked only at much higher levels of I_HVA_ (Figure 4F). The specific levels of I_HVA_ or I_LVA_ required for optimal locking of action potentials to synaptic inputs depend, however, on the intensity and frequency of the stimulus, such that no one set of conductances is optimal for all conditions. In addition, at synapses such as the calyx of Held, the amount of neurotransmitter release and the recovery of the readily releasable pool of neurotransmitter change as a function of firing rate of the presynaptic AVCN cell (Wang and Kaczmarek, 1998; Hermann et al., 2007; Lorteije et al., 2009). Thus, altering potassium current by protein phosphorylation of other second messenger pathways may allow a neuron to adapt appropriately to different patterns and amplitudes of synaptic inputs.

## Kv3 and Kv2 Channels Are Required for High Rates of Firing

The canonical high-voltage activated potassium channels that are required for rapid firing in many auditory brainstem neurons are Kv3 channels, particularly Kv3.1 and Kv3.3 (Kaczmarek and Zhang, 2017). These are expressed in the cochlear nucleus, MNTB, superior olive, and inferior colliculus, as well as many fast-firing neurons in other parts of the adult nervous system (Perney et al., 1992; Perney and Kaczmarek, 1997; Grigg et al., 2000). Under certain circumstances, Kv2.2 can also adopt this role to permit neurons such as those of the MNTB to fire at rates greater than 100 Hz (Steinert et al., 2008, 2011).

The best-studied Kv3 family member is Kv3.1b, which is expressed in the soma of the postsynaptic principal MNTB neurons as well as in the calyx of Held presynaptic terminals (Li et al., 2001; Macica and Kaczmarek, 2001; Elezgarai et al., 2003; Song et al., 2005; Choudhury et al., 2020). Under normal conditions, the voltage-dependence of Kv3.1b channels matches that described above for I_HVA_ currents. As a cell is progressively depolarized, currents begin to appear at potentials of ∼−15 to −10 mV and 50% activation occurs at ∼+15 mV (Luneau et al., 1991; Brown et al., 2016). The Kv3.1b current activates very rapidly during the upstroke of an action potential. A current with these characteristics is present in patch clamp recordings of MNTB neurons (Wang et al., 1998).

Genetic and pharmacological experiments, as well as numerical simulations, using a variety of neurons have shown that Kv3.1b contributes to the rapid repolarization of action potentials that allows them to fire at rates of several hundred Hz (Kaczmarek and Zhang, 2017). In MNTB neurons, genetic deletion of the *Kv3.1* gene does not alter total levels of potassium current (Macica et al., 2003; Choudhury et al., 2020), because of a compensatory increase in Kv3.3 current, with no change in levels of Kv3.3 protein (Choudhury et al., 2020). As a result, the extent to which Kv3.1 knockout alters characteristics of the currents and the ability of MNTB neurons to be driven at high rates depends on experimental factors such as animal strain and recording temperature (Macica et al., 2003; Choudhury et al., 2020).

Kv3.3 channels are also widely expressed in the auditory brainstem (Li et al., 2001). Their conducting properties are in general similar to those of Kv3.1 channels in that they produce I_HVA_ currents that repolarize action potentials (Kaczmarek and Zhang, 2017). Unlike Kv3.1, however, Kv3.3 channels inactivate during sustained depolarization lasting tens to hundreds of milliseconds. Moreover, the cytoplasmic C-terminus of Kv3.3 is larger than that of other members of the Kv3 family. This cytoplasmic region binds several proteins that directly nucleate actin filaments, including Hax-1 and the Arp2/3 complex. As a result, when Kv3.3 channels are inserted into the plasma membrane, they are capable of triggering a dense subcortical actin network (Zhang et al., 2016). Both the cellular and subcellular distribution of Kv3.3 is distinct from that of Kv3.1. At the cellular level, both Kv3.3 and Kv3.1 are expressed in neurons of the AVCN and the MNTB, but only Kv3.3 is found in neurons of the LSO and MSO (Perney and Kaczmarek, 1997; Li et al., 2001). At the subcellular level, Kv3.1 localizes to the “back” face of the terminals (calyces of Held) of AVCN globular bushy cells (Elezgarai et al., 2003), while Kv3.3 localizes to the presynaptic membrane facing the postsynaptic neurons, which is characterized by a dense subcortical actin network (Zhang et al., 2016; Kaczmarek et al., 2019). While immunostaining suggests that, within the MNTB itself, Kv3.3 is largely confined to the presynaptic terminals of AVCN neurons (Zhang et al., 2016), genetic deletion of either Kv3.1 or Kv3.3 does not reduce total potassium current in MNTB neurons, presumably because of compensatory changes in expression of the other subunit (Macica et al., 2003; Choudhury et al., 2020). In contrast, genetic knockout of Kv3.3 reduces potassium current in LSO neurons and severely impairs their ability to fire at high rates (Choudhury et al., 2020).

The importance of Kv3.3 for the discrimination of the source of a sound that is required for the cocktail party effect has come from studies of patients with SCA13, which is caused by mutations in in KCNC3, the human gene encoding Kv3.3 (Zhang and Kaczmarek, 2016). Kv3.3 channels are particularly abundant in cerebellar Purkinje cells and these mutations produce either early-onset or late-onset cerebellar degeneration. As noted earlier, however, late-onset SCA13 patients are completely unable to resolve interaural timing or intensity differences, even decades before they develop any detectable motor symptoms (Middlebrooks et al., 2013).

Kv2.2 channels, unlike Kv3.1 and Kv3.3, do not produce true I_HVA_ currents in that they begin to activate even with small depolarizations from the resting potential and, in MNTB neurons, are already half-activated at ∼−10 mV (Johnston et al., 2008b; Tong et al., 2013). At this potential, the Kv3 channels are just beginning to activate during an action potential (Kanemasa et al., 1995). Although they activate more slowly than Kv3 channels, under appropriate conditions, Kv2.2 can contribute to the repolarization of action potentials (Johnston et al., 2008b; Tong et al., 2013). They can therefore be considered to be hybrid I_HVA_-I_LVA_ channels, and they will be discussed again in a later section.

## Short-Term Modulation of I_HVA_ Channels

The relative contribution of Kv2 and Kv3 family channels to overall current is subject to ongoing modulation by the auditory environment. A change in potassium currents can adapt a neuron to respond appropriately to different frequencies, intensities or patterns of stimulation. The matching of potassium currents to patterns of synaptic inputs may provide an explanation for why in almost every auditory brainstem nucleus, the pattern of expression of potassium channels differs from cell-to-cell and that the level of potassium currents is subject to continual modification by the auditory environment. For example, in common with a subset of other channels in other auditory nuclei, Kv3.1b is expressed along the tonotopic gradient in the MNTB (Figure 5A). Highest levels are found in neurons in the medial, high frequency aspect of the MNTB (Li et al., 2001; von Hehn et al., 2004; Brew and Forsythe, 2005; Strumbos et al., 2010a).

Auditory brainstem neurons and, in particular, neurons of the MNTB and AVCN, have provided key findings on how potassium channels are regulated by incoming stimuli. For example, it is now apparent that Kv3.1 channels in such neurons are regulated over multiple time scales, ranging from tens of seconds to months following changes in auditory inputs, and this will be described in more detail below. There are two ways in which current amplitude can be modulated within a cell. First, rapid changes in potassium currents (occurring in seconds to tens of seconds) can be produced by posttranslational modifications such as phosphorylation of the channel protein. Second, the amount of channel protein in the plasma membrane can be changed, as a result of changes in transcription, translation and/or trafficking into the plasma membrane.

## Phosphorylation of Kv3.1b Channels

Even fundamental aspects of a channel such as its voltage-dependence can be altered by phosphorylation. For example, casein kinase 2, often considered a constitutively active kinase, can adjust the voltage dependence of Kv3.1 current in MNTB neurons (Macica and Kaczmarek, 2001). As stated earlier, Kv3.1 channels are I_HVA_ channels that normally generate significant current at potentials positive to ∼−10 mV. In response to inhibitors of casein kinase 2, however, Kv3.1 currents begin to activate at ∼−40 mV, much closer to the resting potential, effectively turning them into I_LVA_ channels (Kanemasa et al., 1995; Macica et al., 2003). The specific sites on Kv3.1 that are modified by this enzyme are, however, not yet known, nor is it understood under what biological conditions Kv3.1 phosphorylation by casein kinase 2 is altered. The actions of casein kinase 2 can also be mimicked by a novel class of imidazolinedione compounds that convert Kv3.1 currents into I_LVA_-like currents (Taskin et al., 2015; Brown et al., 2016; El-Hassar et al., 2019).

A much clearer biological role for phosphorylation of Kv3.1 has been found for protein kinase C (PKC). There exist two splice variants of the Kv3.1 gene, Kv3.1a and Kv3.1b, which differ only in the presence of a long cytoplasmic C-terminal domain in the Kv3.1b isoform. This longer cytoplasmic domain provides an additional phosphorylation site (serine 503) for PKC. When this site is phosphorylated, the amplitude of Kv3.1b currents is partially suppressed (Figure 6A; Kanemasa et al., 1995; Macica et al., 2003). In auditory neurons, Kv3.1a dominates early in development and Kv3.1b becomes the dominant form after the onset of hearing (Perney et al., 1992; Perney and Kaczmarek, 1997; Liu S.J. and Kaczmarek, 1998a).

A change in the ambient auditory environment, as occurs when a person (or a rat) moves from a quiet setting to a cocktail party situation, produces a change in the phosphorylation of Kv3.1 at residue serine 503 and in I_HVA_ current amplitude. When rats are maintained in a quiet environment, Kv3.1b channels in AVCN and MNTB neurons, as well as in the calyxes of Held, are highly phosphorylated at this site (Song et al., 2005). In response to a physiological increase in sound levels (free-field click trains at 600 Hz, 70 dB sound pressure level (SPL) for 5 min), comparable to a “cocktail party” sound environment, Kv3.1b undergoes dephosphorylation at serine 503 (Figures 6B,C).

Experiments *in vitro* with MNTB brain slices confirmed that Kv3.1b channels are highly phosphorylated in the absence of stimulation, but are dephosphorylated within seconds upon stimulation of the input from the AVCN at 600 Hz (Figures 6D,E; Song et al., 2005). Consistent with the fact that phosphorylation at serine 503 suppresses Kv3.1b current, such stimulation increased I_HVA_ current and increased the ability of the principal neurons of the MNTB to fire action potentials at higher rates of stimulation by intracellular current pulses. Pharmacological and co-immunoprecipitation experiments revealed that the PKC-δ isoform selectively contributes to the basal phosphorylation of the serine 503 site, and that its dephosphorylation during stimulation of the input to the MNTB is mediated by protein phosphatases PP1/PP2A (Song et al., 2005; Song and Kaczmarek, 2006).

As stated above, levels of Kv3.1b channels vary along the tonotopic axis of the MNTB with highest levels in neurons in the medial aspect of the MNTB where neurons preferentially respond to high frequency sounds (Li et al., 2001; von Hehn et al., 2004; Brew and Forsythe, 2005; Strumbos et al., 2010a). It appears that the effect of this gradient of Kv3.1b protein on I_HVA_ current may be further enhanced by phosphorylation. When pharmacological agents are used to maximally stimulate serine 503 phosphorylation, a clear tonotopic gradient of phosphorylation is observed along the lateral-medial axis of the MNTB, exactly matching that of total Kv3.1b protein (Figure 5B; Song et al., 2005). In the absence of stimulation, however, levels of serine 503-phosphorylated Kv3.1b are uniform across this axis. Thus, the proportion of phosphorylated (i.e., suppressed) Kv3.1 channels is greatest in neurons at the lateral low-frequency end of the nucleus and lowest at the medial high-frequency end (Figure 5C; Song et al., 2005). Whether this difference in phosphorylation at different ends of the MNTB reflects a gradient of expression of PKC, PP1/P2A or some other regulator of signaling pathways is not yet known. Nevertheless, this finding suggests that under such basal conditions the gradient in I_HVA_ current is greater than that of levels of Kv3.1b itself, but that this can be modified by auditory inputs over a time course of seconds to minutes.

## Phosphorylation of Kv3.3 Channels

The Kv3.3 channel, which is co-expressed with Kv3.1 in the presynaptic calyx of Held, is also regulated by PKC (Desai et al., 2008; Zhang et al., 2016). In contrast to Kv3.1b, however, the major phosphorylated residues are located on the cytoplasmic N-terminus, and phosphorylation of these sites increases rather than decreases current (Desai et al., 2008). Whether these sites on Kv3.3 are modified during changes in the auditory environment and how they impact the function of the MNTB are not yet known.

## Rapid Regulation of Kv2.2 Channels in MNTB Neurons

Numerous numerical computations of the gating of Kv3 channels have shown that these channels are essential for neurons to fire at high rates. This is because their rapid deactivation minimizes the relative refractory period that follows an action potential. Indeed, deactivation is so rapid that a unique gating process ensures complete repolarization; Kv3.1 generates a resurgent potassium current during the falling phase of an action potential that provides the repolarization drive to terminate each spike in a train (Labro et al., 2015). Nevertheless, it appears that Kv3 channels sometimes delegate some of their role in repolarization to Kv2.2 channels, perhaps when firing rates are low for a sustained period. Like Kv3.1, the “hybrid I_HVA_-I_LVA_” Kv2.2 channels are expressed in a gradient along the tonotopic axis of the MNTB, but this gradient is in the opposite direction from that of Kv3.1, with highest levels in the lateral low-frequency neurons (Johnston et al., 2008b; Tong et al., 2013). They contribute to the hyperpolarization between action potentials during repetitive firing such that genetic elimination of Kv2.2 reduces the number of action potentials that can be evoked by repetitive stimulation (Johnston et al., 2008b; Tong et al., 2013). Stimulation of presynaptic inputs to MNTB at low rates, at or below those encountered *in vivo* in silence (10 Hz-150 Hz stimulation Brownell, 1975; Hermann et al., 2007; Kopp-Scheinpflug et al., 2008), suppresses Kv3.1 currents (Steinert et al., 2008) while increasing the amplitude of Kv2.2 currents (Steinert et al., 2011). The mechanism of this increase has been shown to require the release of nitric oxide (NO), and activation of the cyclic GMP-dependent protein kinase and PKC (Steinert et al., 2008, 2011). Although it is known that phosphorylation of specific sites on the closely related Kv2.1 potassium channel in other cells influences its biophysical properties (Ikematsu et al., 2011), and its insertion into the plasma membrane (He et al., 2015), the specific sites on Kv2.2 required for its recruitment in MNTB neurons are yet known.

## Activity-Dependent Changes in Expression of Potassium Channel Proteins

The phospho-specific immunostaining techniques used to examine Kv3.1b in the experiments described above allowed changes to be detected within 60 s of stimulation (Song and Kaczmarek, 2006). Changes in phosphorylation state can, however, occur within less than a second of stimulation, so changes in I_HVA_ current in response to auditory input may occur more rapidly than was detected in those experiments. In addition to these rapid changes, there is also a much slower change in the levels of Kv3.1b channel protein in the plasma membrane that is also brought about by incoming sounds. The difference in levels of Kv3.1b protein between the medial high-frequency and the lateral low-frequency MNTB neurons is maintained by ongoing auditory stimulation. This gradient is absent in mice that undergo hearing loss because of cochlear hair cell degeneration (von Hehn et al., 2004; Leao et al., 2006b). In the C57BL/6 strain of mice, which undergoes age-related hearing loss, the Kv3.1b gradient in the MNTB is present for the first few months after birth, but is lost by 6 months of age (von Hehn et al., 2004).

Exposure of normal rats or mice to sound stimuli similar to those that produce Kv3.1b dephosphorylation also triggers the synthesis of new Kv3.1b protein and, depending on the frequency distribution of the sound, can alter the tonotopic gradient (Leao et al., 2010; Strumbos et al., 2010a,b). Changes in levels of Kv3.1b protein in MNTB neurons can be detected within 20 min to one hour after the onset of the sound stimulus. One interesting aspect of these experiments is that levels of Kv3.1b in neurons outside of the tonotopic region targeted by the auditory stimulus may decrease during the same time period (Strumbos et al., 2010b). This observation suggests the existence of mechanisms that regulate the response of the MNTB globally, perhaps by sideband inhibition or other mechanisms that govern lateral interactions among neurons in this nucleus (Kaczmarek, 2019).

## The FMRP Protein Regulates Levels of Kv3.1 Channels in Response to Stimulation

A key mechanism that plays a role in stimulating the production of new Kv3.1b channels in response to auditory stimulation is the FMRP pathway. FMRP is an mRNA-binding protein that is required for activity-dependent translation of mRNAs for a large subset of proteins (De Rubeis and Bagni, 2010; Darnell et al., 2011; Richter et al., 2015). Human patients lacking FMRP have Fragile X syndrome, the leading known inherited cause of autism, and loss of FMRP leads to hypersensitivity to auditory stimuli and impairs the ability to localize sounds in space (Hall et al., 2009; Rotschafer and Razak, 2014).

**FIGURE 7:**
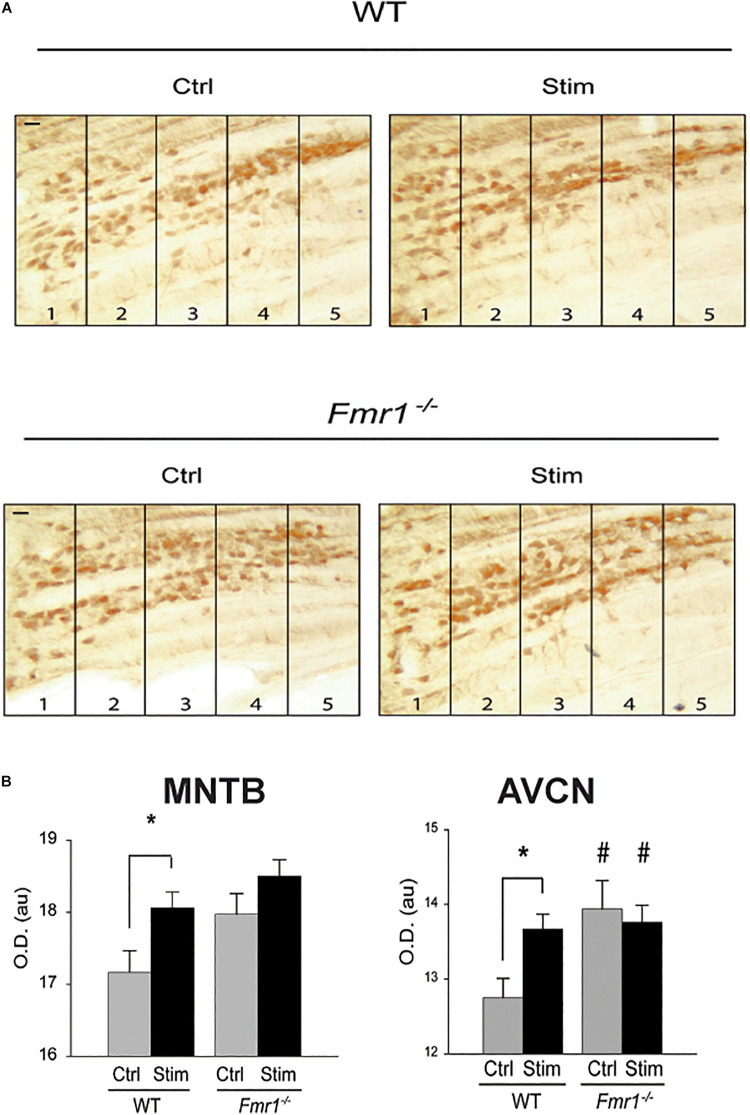
FMRP is required for sound-induced increases in Kv3.1b protein in MNTB. **(A)** Representative sections immunolabeled for total Kv3.1b protein in wild type mice or those in which the gene for FMRP was deleted (*Fmr1*^–/–^). Animals were maintained in silence (Ctrl) or exposed to acoustic stimulation for 30 min (Stim, 32 kHz tones amplitude-modulated at 380-420 Hz, 65 dB). Numbers refer to lateral-to-medial tonotopic zones 1-5 as in Figure 5. **(B)** Quantification of changes in Kv3.1b in MNTB and AVCN demonstrates that stimulation increases levels of protein in the wild type mice but not those lacking FMRP, which have uniformly higher levels of Kv3.1b in the absence of stimulation (*p = 0.02; ^#^*p* = 0.04 compared to wild type controls) (Strumbos et al., 2010a).

While FMRP is now known to control a variety of biological processes, one of the ways it influences protein synthesis is by binding its target mRNAs and suppressing their translation. Subsequent neuronal stimulation may alleviate this block of translation, leading to enhanced synthesis of the protein. Messenger RNA for Kv3.1 was one of the very first described targets for FMRP (Darnell et al., 2001, 2011; Strumbos et al., 2010a). Consistent with this “canonical” role for FMRP in regulating the expression of this channel, mice lacking FMRP have elevated levels of Kv3.1 protein and I_HVA_ currents in MNTB neurons (Strumbos et al., 2010a). The tonotopic gradient of Kv3.1 is absent in these animals (Figure 7A), and auditory stimulation has no effect in further enhancing levels of Kv3.1 in either the MNTB or AVCN (Figure 7B), suggesting that rates of translation are maximal in the absence of FMRP (Strumbos et al., 2010a). While it is likely that lack of FMRP results in loss of tonotopy and activity-dependent translation for many of the other proteins whose mRNAs are targets of this mRNA-binding protein, this has not been tested directly (McCullagh et al., 2020).

## Regulation of Transcription of I_HVA_ Channel mRNAs by CREB

Rapid adjustment of the levels of I_HVA_ currents by activation of protein kinases, and potentially the slower changes produced by activity-dependent synthesis or insertion into the plasma membrane may play a role in the cocktail party effect by adjusting firing patterns to maximize the extraction of aspects of a sound required for localization in space. The fact that individuals vary considerably in their ability to localize sounds may result, however, from differences in even longer-term mechanisms, specifically regulation of transcription, which influences levels of ion channel mRNA in each neuron. The gene for Kv3.1 has a cyclic AMP/Ca2^+^-response element (CRE) upstream of the start of transcription (Gan et al., 1996; Gan and Kaczmarek, 1998). Transcription of the Kv3.1 gene is triggered when stimulation of neurons elevates cytoplasmic cyclic AMP or Ca^2+^ levels. These cause the phosphorylation of the transcription factor CREB (Cyclic AMP/Ca^2+^-Responsive Element-Binding protein) (Gonzalez and Montminy, 1989; Dash et al., 1991), which then binds the CRE and activates synthesis of Kv3.1 mRNA (Gan et al., 1996). Depolarization of inferior colliculus neurons for six hours (Liu S.Q. and Kaczmarek, 1998b) or of long-term cultures of MNTB neurons for several days (Tong et al., 2010), using a high potassium external solution, has been shown to increase I_HVA_ currents and levels of mRNA for Kv3.1 and Kv3.3 channel subunits, respectively.

**FIGURE 8:**
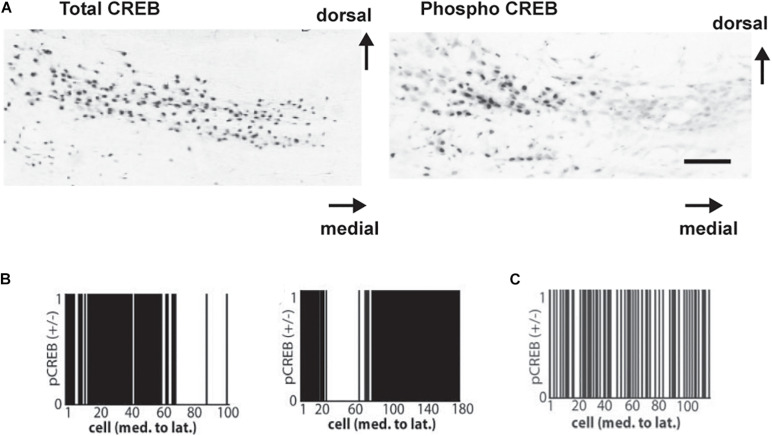
**(A)** Coronal sections showing immunoreactivity for CREB (*left*) and for pCREB (*right*) in mice with good hearing (8-month-old CBA/J strain). Scale bar, 250 μm. **(B)** Clustered distribution of nuclear pCREB immunoreactivity in all cells across the medial-lateral axis for two normal hearing mice. Cells positive for pCREB were assigned a value of 1, and those lacking pCREB were assigned a value of 0. **(C)** As **(B)** but showing a random distribution of pCREB immunoreactivity in a hearing-impaired mouse (6 month old C57BL/6 strain is shown) (von Hehn et al., 2004).

To examine the role of CREB phosphorylation *in vivo*, immunostaining for phosphorylated CREB (pCREB) in the MNTB has been used to determine which neurons are likely to be actively transcribing mRNA for Kv3.1 and other genes regulated by a CRE (von Hehn et al., 2004; Figure 8A). These experiments have demonstrated that transcription, like Kv3.1 protein synthesis and phosphorylation, is actively controlled by auditory inputs. All neurons in the MNTB appear to have similar levels of CREB itself. The phosphorylation of CREB, however, appears to be an “all or none” event that occurs in subsets of neurons clustered at different locations along the tonotopic axis. Because the location of these clusters along the axis varies from animal to animal in fixed tissue, it is likely that these patterns reflect differences in incoming auditory inputs at the time of fixation. More significantly, these clusters are completely absent in hearing impaired mice (von Hehn et al., 2004; Figures 8B,C).

## Kv1 Family I_LVA_ Channels Activate Close to the Resting Membrane Potential

Several different classes of potassium channels contribute to I_LVA_ currents in auditory brainstem neurons. In general, I_LVA_ potassium currents endow neurons with a low input resistance and fast time constant and, in the auditory brainstem, are prominent in neurons that lock their action potential precisely to incoming auditory stimuli, such as bushy cells of the AVCN, MNTB neurons and octopus cells in the posteroventral cochlear nucleus (Oertel, 1983; Smith and Rhode, 1987; Manis and Marx, 1991; Brew and Forsythe, 1995; Oertel et al., 2008; Rusznak et al., 2008). Because these currents start to activate significantly with small depolarizations from the resting potential, they limit the firing of action potentials in response to sustained depolarization or prolonged postsynaptic potentials (Manis and Marx, 1991; Schwarz and Puil, 1997; Rothman and Manis, 2003; Cao et al., 2007).

I_LVA_ currents also play a key role in neurons of the LSO and the MSO (Barnes-Davies et al., 2004; Svirskis et al., 2004; Mathews et al., 2010; Fischer et al., 2018; Nabel et al., 2019). In the MSO, I_LVA_ currents are present in proximal dendrites and the soma, and their activation by excitatory synaptic inputs in the dendrites shortens the duration of the excitatory postsynaptic potentials (EPSPs) as they propagate toward the soma (Mathews et al., 2010). This feature contributes to the ability of MSO neurons to resolve differences of the order of tens of microseconds in the time or arrival of binaural inputs. In the LSO, there is a tonotopic gradient of I_LVA_ currents, with high levels of I_LVA_ in neurons in the lateral, low frequency limb of this nucleus (Barnes-Davies et al., 2004). As in the AVCN and MNTB, this limits the firing of action potentials in response to sustained depolarization and serves to preserve timing information (Remme et al., 2014).

The dominant and best studied I_LVA_ currents in MNTB and LSO neurons are produced by the Kv1.1 and Kv1.2 voltage-dependent subunits (Dodson et al., 2002, 2003; Barnes-Davies et al., 2004; Gittelman and Tempel, 2006). These are expressed ubiquitously throughout the central and peripheral nervous system. In principal neurons of the MNTB, heteromeric Kv1.1/Kv1.2 channels are localized to the initial segment of the axon of postsynaptic neurons, where they provide a dominant component of the I_LVA_ current (Dodson et al., 2002). The activation of these channels ensures that a synaptic input or a sustained depolarization produces only a single action potential that is faithfully locked to the onset of the stimulus (Oertel, 1983; Smith and Rhode, 1987). This mode of response is essential for accurate localization of sounds in space. Thus, knockout of the gene for the Kv1.1 channel in mice increases the latency and jitter of sound-evoked action potentials in MNTB neurons (Kopp-Scheinpflug et al., 2003a; Gittelman and Tempel, 2006) and renders the mice unable to localize sounds (Karcz et al., 2011; Robbins and Tempel, 2012).

There is evidence that several other potassium channels may contribute to the I_LVA_ currents of MNTB postsynaptic neurons. These include the voltage-dependent channel subunits Kv1.6 (Dodson et al., 2002) and Kv11 (Hardman and Forsythe, 2009), as well as K_Na_1.1 and K_Na_1.2 (also termed Slack and Slick), potassium channels activated by elevations of intracellular sodium (K_Na_ channels) (Bhattacharjee et al., 2002, 2005; Yang et al., 2007a).

The complement of I_LVA_ channels in the presynaptic calyx of Held terminals is slightly different from those in the postsynaptic cells. Kv1.2 homomers have been documented on the membrane of the presynaptic axons (Dodson et al., 2003). Kv1.3 channels are also localized to the plasma membrane of the presynaptic terminal and to small intracellular vesicles in the cytoplasm of the terminal (Gazula et al., 2010). Within this terminal, the role of I_LVA_ currents is likely to differ from in the postsynaptic cells because the firing pattern of the terminals is driven by action potentials generated at the cell body of the AVCN bushy cell. One such role for the Kv1.2 channels is to ensure that each incoming action potential from the axon triggers only a single spike at the terminal itself (Dodson et al., 2003).

Compared to the I_HVA_ channels, much less is known about factors that modulate Kv1 family channels in response to changes in the sound environment. These channels all have documented phosphorylation sites and experiments carried out largely using non-excitable cells suggest that their current amplitude and trafficking can be regulated by second messenger pathways (Vacher and Trimmer, 2011). For example, the amplitude of Kv1.1 currents has been reported to be regulated by the cyclic-AMP-dependent protein kinase (Winklhofer et al., 2003). Phosphorylation of serine 446 in Kv1.1 modulates its association with auxiliary subunits (Singer-Lahat et al., 1999). Phosphorylation of Kv1.2 regulates its intracellular trafficking (Yang et al., 2007b). PKC also regulates Kv1.1 through a mechanism that does not require its consensus phosphorylation sites (Boland and Jackson, 1999). Whether any of these mechanisms are engaged by stimulation of auditory neurons is, however, unknown.

## Potential Contributions of K_*Na*_ Channels to I_LVA_ Currents

The K_Na_1.1 and K_Na_1.2 channels are widely expressed in the nervous system, including auditory brainstem neurons (Bhattacharjee et al., 2002, 2003). Based on what is known about their biochemical and biophysical properties, it is possible to make some tentative predictions about how their regulation contributes to I_LVA_ currents in MNTB neurons and related cells. For example, the higher levels of firing induced by high intensity sounds will elevate intracellular sodium levels by sodium entry both through voltage-dependent sodium channels and ionotropic glutamate receptors on MNTB neurons. These would be expected to increase the activity of the sodium sensitive K_Na_ channels (Kaczmarek, 2013; Kaczmarek et al., 2017). Using brain slice preparations, it has been shown that drugs that promote the opening of K_Na_ channels increase the fidelity with which MNTB neurons lock their action potentials to a stimulus train (Yang et al., 2007a).

Phosphorylation also plays a direct role in regulating the amplitude of K_Na_ currents. For example, the K_Na_1.1 channel has a serine residue (serine 407 in the rat channel) that, when phosphorylated by PKC, increases current amplitude (Santi et al., 2006; Barcia et al., 2012). If an increase in sound intensity in the physiological range causes dephosphorylation of this K_Na_1.1 residue, as occurs for serine 503 in Kv3.1, this would enhance I_LVA_ current and aid temporal accuracy of firing. Such short term increases in I_LVA_ current in brainstem neurons could potentially contribute to improving the discrimination of sounds in a noisy environment, as occurs in the cocktail party effect (Zion Golumbic et al., 2013). Speculations on increases in I_LVA_ are limited by the fact that the effects of PKC activation on K_Na_1.1 channels differ from those on K_Na_1.2 channels or K_Na_1.1/K_Na_1.2 heteromeric channels (Santi et al., 2006). Human mutations in K_Na_1.1 that increase current amplitude result in intellectual deficits so severe that they preclude the characterization of auditory function (Kim and Kaczmarek, 2014).

## Direct Effects of FMRP Binding to I_LVA_ Channel Subunits

As described earlier, patients lacking the RNA-binding protein FMRP, are impaired in their ability to localize sounds and suffer from hyperacusis. At least part of this disability may result not only from the effects of FMRP on translation of mRNAs but because FMRP binds directly to some of the ion channels that provide I_LVA_ currents (McCullagh et al., 2020). Both Kv1.2 and K_Na_1.1 channels have been found to exist in a protein complex with FMRP (Brown et al., 2010; Zhang et al., 2012; Yang et al., 2018). Interestingly, binding of FMRP to Kv1.2 has been found to require prior phosphorylation of the channel at serine residues in its cytoplasmic C-terminal domain (Yang et al., 2018). This interaction enhances the activity of Kv1.2 channels. Similarly, the interaction of FMRP with the cytoplasmic C-terminus of K_Na_1.1 potently stimulates channel activity (Brown et al., 2010; Zhang et al., 2012). Consistent with these findings, the I_LVA_ component of potassium current is substantially reduced in MNTB neurons in a mouse model of Fragile X syndrome in which the gene for FMRP has been deleted (Brown et al., 2010; El-Hassar et al., 2019).

Like the mRNAs for the I_HVA_ channels Kv3.1 and Kv3.3, the mRNAs for Kv1.2 and K_Na_1.1 are targets of FMRP (Darnell et al., 2011). One might expect, therefore, that in addition to the direct effects of loss of FMRP-binding on channel activity, the rates of synthesis of these two proteins in response to changes in the auditory environment may be compromised in animals and humans lacking FMRP. Nevertheless, whether I_LVA_ currents are altered by auditory stimuli, and the full time-course and mechanisms of such changes, are yet to be investigated.

A study has compared the relative amplitudes of I_LVA_ and I_HVA_ components of potassium current in MNTB neurons from wild type mice and those lacking FMRP (El-Hassar et al., 2019). Consistent with the data described in the preceding sections, the ratio of I_HVA_ to I_LVA_ is enhanced in the FMRP knockout animals. Because increases in I_HVA_ enhance high frequency firing while I_LVA_ currents are required for temporal accuracy, this change in both components is consistent with both the hypersensitivity of Fragile X patients to loud sounds and their inability to localize sounds in space. Pharmacological agents that alter the voltage dependence of Kv3.1 channels, converting them from I_HVA_ to I_LVA_ channels, may provide a potential direction for helping to ameliorate the auditory phenotype of Fragile X patients (El-Hassar et al., 2019).

## Other Channels That Modulate Response Properties

Several other types of potassium channels are expressed in the MNTB of different species and at different times in development. These include Kv4.3 subunits, which have been reported in the MNTB of mice but not that of rats (Johnston et al., 2008a). These subunits generate rapidly inactivating “A-type” potassium currents, which could potentially influence the timing of neuronal responses, but their precise role is not understood. Immunoreactivity for a second inactivating potassium channel subunit, Kv3.4, has been reported in the calyx of Held presynaptic terminals (Ishikawa et al., 2003), but mRNA for this subunit is absent in the MNTB (Choudhury et al., 2020), suggesting that Kv3.4 could be synthesized in the AVCN and then transported to the calyces. Because Kv3.4 is expressed in all major fiber tracts in the developing brain, it is possible that this subunit has a role in the navigation of the fibers of AVCN neurons early in development (Huang et al., 2012).

Finally, there exists a class of potassium currents that maintain the membrane potential of a neuron close its normal negative value (∼−45 to −75 mV). The basic concept of a leak potassium channel arose from finding that pharmacological block of most of the known categories of potassium channels does not abolish this resting potential. Thus, leak channels have a fixed open probability that does not change with voltage or rapid changes in intracellular Ca^2+^ or Na^+^. Nearly all numerical simulations of the firing patterns of neurons incorporate such a voltage-independent leak current that reverses at a negative potential (Wang et al., 1998; Rothman and Manis, 2003; Song et al., 2005; Kaczmarek, 2012). Because leak potassium channels are open at all voltages, changes in such leak currents could potentially alter all aspects of neuronal firing, including the resting potential, the time constant and the threshold and height of action potentials. In many cases, however, the magnitude of leak currents is smaller than that of the other “active” potassium currents, which dominate these aspects. The subfamily of two pore domain potassium channels (K_2P_, also termed KCNK) contains fifteen members, and has been found to give rise to leak-type K^+^ channels. In contrast to other potassium channels, in which four α-subunits assemble to form a functional channel, K_2P_ channels assemble as dimers (Niemeyer et al., 2016; Zuniga and Zuniga, 2016).

Expression of the mRNAs for several K_2P_ subunits has been documented in the auditory brainstem. These include K_2P_15 (also termed TASK5), K_2P_1 (TWIK-1), K_2P_12 (THIK-2), K_2P_6 (TWIK-2), K_2P_2 (TREK-1), K_2P_10(TREK-2), K_2P_4 (TRAAK), and K_2P_9 (TASK-3) in the rat cochlear nucleus (Holt et al., 2006). MNTB neurons express mRNA for K_2P_1 (Kaczmarek et al., 2005), and the properties of the leak currents in MNTB neurons have been found to match that of K_2P_1 (Berntson and Walmsley, 2008). Of particular interest is the K_2P_15 (TASK5) subunit, which, except for a subset of cerebellar and olfactory neurons, is expressed almost exclusively in central auditory pathways (Karschin et al., 2001; Ehmann et al., 2013). When expressed by itself in heterologous expression systems, K_2P_15 fails to generate currents (Karschin et al., 2001), suggesting functional channels in neurons represent a heteromer of K_2P_15 with another K_2P_ subunit. Deafening by cochlear ablation produces a large sustained reduction in expression of K_2P_15 in brainstem neurons and inferior colliculus, and also decreases expression of several other K_2P_ subunits (Holt et al., 2006; Cui et al., 2007; Dong et al., 2009). Acute shRNA-mediated knockdown of K_2P_15 in auditory produces a depolarization of the resting membrane potential, increasing the width and latency of action potentials and enhanced firing in globular bushy cells of the cochlear nucleus, with smaller effects in MNTB neurons, which have lower K_2P_15 expression (Saher, 2020). These findings suggest that signaling pathways that modify K_2P_ currents in auditory neurons could have major effects on their intrinsic excitability.

## Conclusion

Research over the past two decades has amply demonstrated that the intrinsic excitability of neurons responsible for the very early stages of auditory processing are not fixed. The importance of the correct balance of ion channels is evident from human conditions such as SCA13 and Fragile X syndrome, which do not result in deafness but severely impair the ability to distinguish sounds in a noisy environment. Even rapid changes in the auditory environment can produce rapid and reversible posttranslational modifications of channels expressed in auditory brainstem nuclei such as the MNTB. Such rapid changes in excitability are certain to contribute to an alteration in the way in which sounds are processed when a person moves from a quiet environment to a noisy one, such as one in which the cocktail party effect is manifest. Longer-term mechanisms that depend on incoming sounds are required to maintain the appropriate balance of different ion channels. Some of these mechanisms involve the transcription and translation of mRNAs for channel subunits and are required for the correct distribution of several ion channels along the tonotopic axis of auditory nuclei. Such longer-term changes in ion channels may also contribute to experience-dependent differences in the auditory abilities of individuals, for example, the superior ability of orchestra conductors to localize sounds (Münte et al., 2001; Nager et al., 2003). Nevertheless, because of their experimental tractability, rats and mice have been the major species used for the study of potassium channels properties in auditory neurons. Future work will require the determination of how many of the findings can be generalized to other species, particularly those with hearing that more closely matches that of humans.

The correction of the abnormalities in auditory processing related to ion channels in humans may involve both pharmacological approaches to alter channel activity and genetic approaches that correct the underlying defects. For example, pharmacological agents that alter the gating of Kv3 family channels have the potential to correct the abnormal firing patters of auditory brainstem neurons in Fragile X syndrome (El-Hassar et al., 2019). Changing the levels of expression of either wild type or mutant ion channels can be achieved using antisense oligonucleotides, and these may come to be used as a therapy for genetic diseases such as SCA13 (Bushart and Shakkottai, 2019).

## Author Contributions

Both authors equally contributed to the drafting and writing of the final version of the manuscript and read and approved the final version of the manuscript.

## Conflict of Interest

The authors declare that the research was conducted in the absence of any commercial or financial relationships that could be construed as a potential conflict of interest. The handling editor is currently organizing a Research Topic with one of the authors LK.

## References

[B1] Alexander S. P. H. Mathie A. Peters J. A. Veale E. L. Striessnig J. Kelly E. (2019). THE CONCISE GUIDE TO PHARMACOLOGY 2019/20: ion channels. *Br. J. Pharmacol.* 176(Suppl. 1) S142–S228. 31710715 10.1111/bph.14749 PMC6844578

[B2] Arinami T. Sato M. Nakajima S. Kondo I. (1988). Auditory brain-stem responses in the fragile X syndrome. *Am. J. Hum. Genet.* 43 46–51. 3376943 PMC1715284

[B3] Banks M. I. Smith P. H. (1992). Intracellular recordings from neurobiotin-labeled cells in brain slices of the rat medial nucleus of the trapezoid body. *J. Neurosci.* 12 2819–2837. 10.1523/jneurosci.12-07-02819.1992 1351938 PMC6575844

[B4] Barcia G. Fleming M. R. Deligniere A. Gazula V. R. Brown M. R. Langouet M. (2012). De novo gain-of-function KCNT1 channel mutations cause malignant migrating partial seizures of infancy. *Nat. Genet.* 44 1255–1259. 10.1038/ng.2441 23086397 PMC3687547

[B5] Barnes-Davies M. Barker M. C. Osmani F. Forsythe I. D. (2004). Kv1 currents mediate a gradient of principal neuron excitability across the tonotopic axis in the rat lateral superior olive. *Eur. J. Neurosci.* 19 325–333. 10.1111/j.0953-816x.2003.03133.x 14725627

[B6] Baydyuk M. Xu J. Wu L. G. (2016). The calyx of Held in the auditory system: structure, function, and development. *Hear. Res.* 338 22–31. 10.1016/j.heares.2016.03.009 27018297 PMC4967386

[B7] Berntson A. K. Walmsley B. (2008). Characterization of a potassium-based leak conductance in the medial nucleus of the trapezoid body. *Hear. Res.* 244 98–106. 10.1016/j.heares.2008.08.003 18761066

[B8] Bhattacharjee A. Gan L. Kaczmarek L. K. (2002). Localization of the slack potassium channel in the rat central nervous system. *J. Comp. Neurol.* 454 241–254. 10.1002/cne.10439 12442315

[B9] Bhattacharjee A. Joiner W. J. Wu M. Yang Y. Sigworth F. J. Kaczmarek L. K. (2003). Slick (Slo2.1), a rapidly-gating sodium-activated potassium channel inhibited by ATP. *J. Neurosci.* 23 11681–11691. 10.1523/jneurosci.23-37-11681.2003 14684870 PMC6740956

[B10] Bhattacharjee A. Von Hehn C. A. Mei X. Kaczmarek L. K. (2005). Localization of the Na+-activated K+ channel Slick in the rat central nervous system. *J. Comp. Neurol.* 484 80–92. 10.1002/cne.20462 15717307

[B11] Boland L. M. Jackson K. A. (1999). Protein kinase C inhibits Kv1.1 potassium channel function. *Am. J. Physiol.* 277 C100–C110. 10409113 10.1152/ajpcell.1999.277.1.C100

[B12] Borst J. G. Soria van Hoeve J. (2012). The calyx of Held synapse: from model synapse to auditory relay. *Annu. Rev. Physiol.* 74 199–224. 10.1146/annurev-physiol-020911-153236 22035348

[B13] Brew H. M. Forsythe I. D. (1995). Two voltage-dependent K+ conductances with complementary functions in postsynaptic integration at a central auditory synapse. *J. Neurosci.* 15 8011–8022. 10.1523/jneurosci.15-12-08011.1995 8613738 PMC6577951

[B14] Brew H. M. Forsythe I. D. (2005). Systematic variation of potassium current amplitudes across the tonotopic axis of the rat medial nucleus of the trapezoid body. *Hear. Res.* 206 116–132. 10.1016/j.heares.2004.12.012 16081003

[B15] Brown M. R. El-Hassar L. Zhang Y. Alvaro G. Large C. H. Kaczmarek L. K. (2016). Physiological modulators of Kv3.1 channels adjust firing patterns of auditory brain stem neurons. *J. Neurophysiol.* 116 106–121. 10.1152/jn.00174.2016 27052580 PMC4961756

[B16] Brown M. R. Kronengold J. Gazula V. R. Chen Y. Strumbos J. G. Sigworth F. J. (2010). Fragile X mental retardation protein controls gating of the sodium-activated potassium channel Slack. *Nat. Neurosci.* 13 819–821. 10.1038/nn.2563 20512134 PMC2893252

[B17] Brownell W. E. (1975). Organization of the cat trapezoid body and the discharge characteristics of its fibers. *Brain Res.* 94 413–433. 10.1016/0006-8993(75)90226-7 1156852

[B18] Bushart D. D. Shakkottai V. G. (2019). Ion channel dysfunction in cerebellar ataxia. *Neurosci. Lett.* 688 41–48. 10.1016/j.neulet.2018.02.005 29421541 PMC6077100

[B19] Cao X. J. Shatadal S. Oertel D. (2007). Voltage-sensitive conductances of bushy cells of the Mammalian ventral cochlear nucleus. *J. Neurophysiol.* 97 3961–3975. 10.1152/jn.00052.2007 17428908

[B20] Carr C. E. MacLeod K. M. (2010). Microseconds matter. *PLoS Biol.* 8:e1000405. 10.1371/journal.pbio.1000405 20613856 PMC2893944

[B21] Castren M. Paakkonen A. Tarkka I. M. Ryynanen M. Partanen J. (2003). Augmentation of auditory N1 in children with fragile X syndrome. *Brain Topogr.* 15 165–171. 12705812 10.1023/a:1022606200636

[B22] Choudhury N. Linley D. Richardson A. Anderson M. Robinson S. W. Marra V. (2020). Kv3.1 and Kv3.3 subunits differentially contribute to Kv3 channels and action potential repolarization in principal neurons of the auditory brainstem. *J. Physiol.* 598 2199–2222. 10.1113/jp279668 32246836

[B23] Cui Y. L. Holt A. G. Lomax C. A. Altschuler R. A. (2007). Deafness associated changes in two-pore domain potassium channels in the rat inferior colliculus. *Neuroscience* 149 421–433. 10.1016/j.neuroscience.2007.05.054 17884299 PMC2699593

[B24] Darnell J. C. Jensen K. B. Jin P. Brown V. Warren S. T. Darnell R. B. (2001). Fragile X mental retardation protein targets G quartet mRNAs important for neuronal function. *Cell* 107 489–499. 10.1016/s0092-8674(01)00566-9 11719189

[B25] Darnell J. C. Van Driesche S. J. Zhang C. Hung K. Y. Mele A. Fraser C. E. (2011). FMRP stalls ribosomal translocation on mRNAs linked to synaptic function and autism. *Cell* 146 247–261. 10.1016/j.cell.2011.06.013 21784246 PMC3232425

[B26] Dash P. K. Karl K. A. Colicos M. A. Prywes R. Kandel E. R. (1991). cAMP response element-binding protein is activated by Ca2+/calmodulin- as well as cAMP-dependent protein kinase. *Proc. Natl. Acad. Sci. U.S.A.* 88 5061–5065. 10.1073/pnas.88.11.5061 1647024 PMC51807

[B27] De Rubeis S. Bagni C. (2010). Fragile X mental retardation protein control of neuronal mRNA metabolism: Insights into mRNA stability. *Mol. Cell. Neurosci.* 43 43–50. 10.1016/j.mcn.2009.09.013 19837168

[B28] Desai R. Kronengold J. Mei J. Forman S. A. Kaczmarek L. K. (2008). Protein kinase C modulates inactivation of Kv3.3 channels. *J. Biol. Chem.* 283 22283–22294. 10.1074/jbc.m801663200 18539595 PMC2494927

[B29] Dodson P. D. Barker M. C. Forsythe I. D. (2002). Two heteromeric Kv1 potassium channels differentially regulate action potential firing. *J. Neurosci.* 22 6953–6961. 10.1523/jneurosci.22-16-06953.2002 12177193 PMC6757903

[B30] Dodson P. D. Billups B. Rusznak Z. Szucs G. Barker M. C. Forsythe I. D. (2003). Presynaptic rat Kv1.2 channels suppress synaptic terminal hyperexcitability following action potential invasion. *J. Physiol.* 550 27–33. 10.1113/jphysiol.2003.046250 12777451 PMC2343026

[B31] Dong S. Mulders W. H. Rodger J. Robertson D. (2009). Changes in neuronal activity and gene expression in guinea-pig auditory brainstem after unilateral partial hearing loss. *Neuroscience* 159 1164–1174. 10.1016/j.neuroscience.2009.01.043 19356697

[B32] Ehmann H. Hartwich H. Salzig C. Hartmann N. Clement-Ziza M. Ushakov K. (2013). Time-dependent gene expression analysis of the developing superior olivary complex. *J. Biol. Chem.* 288 25865–25879. 10.1074/jbc.m113.490508 23893414 PMC3764792

[B33] Elezgarai I. Diez J. Puente N. Azkue J. J. Benitez R. Bilbao A. (2003). Subcellular localization of the voltage-dependent potassium channel Kv3.1b in postnatal and adult rat medial nucleus of the trapezoid body. *Neuroscience* 118 889–898. 10.1016/s0306-4522(03)00068-x 12732235

[B34] El-Hassar L. Song L. Tan W. J. T. Large C. H. Alvaro G. Santos-Sacchi J. (2019). Modulators of Kv3 potassium channels rescue the auditory function of fragile X mice. *J. Neurosci.* 39 4797–4813. 10.1523/jneurosci.0839-18.2019 30936239 PMC6561694

[B35] Ferron L. (2016). Fragile X mental retardation protein controls ion channel expression and activity. *J. Physiol.* 594 5861–5867. 10.1113/jp270675 26864773 PMC5063927

[B36] Fischer L. Leibold C. Felmy F. (2018). Resonance properties in auditory brainstem neurons. *Front. Cell. Neurosci.* 12:8. 10.3389/fncel.2018.00008 29416503 PMC5787568

[B37] Forsythe I. D. Barnes-Davies M. (1993). The binaural auditory pathway: membrane currents limiting multiple action potential generation in the rat medial nucleus of the trapezoid body. *Proc. Biol. Sci.* 251 143–150. 10.1098/rspb.1993.0021 8096080

[B38] Gan L. Kaczmarek L. K. (1998). When, where, and how much? Expression of the Kv3.1 potassium channel in high-frequency firing neurons. *J. Neurobiol.* 37 69–79. 10.1002/(sici)1097-4695(199810)37:1<69::aid-neu6>3.0.co;2-6 9777733

[B39] Gan L. Perney T. M. Kaczmarek L. K. (1996). Cloning and characterization of the promoter for a potassium channel expressed in high frequency firing neurons. *J. Biol. Chem.* 271 5859–5865. 10.1074/jbc.271.10.5859 8621457

[B40] Gazula V. R. Strumbos J. G. Mei X. Chen H. Rahner C. Kaczmarek L. K. (2010). Localization of Kv1.3 channels in presynaptic terminals of brainstem auditory neurons. *J. Comp. Neurol.* 518 3205–3220. 10.1002/cne.22393 20575068 PMC2894291

[B41] Gittelman J. X. Tempel B. L. (2006). Kv1.1-containing channels are critical for temporal precision during spike initiation. *J. Neurophysiol.* 96 1203–1214. 10.1152/jn.00092.2005 16672305

[B42] Gonzalez G. A. Montminy M. R. (1989). Cyclic AMP stimulates somatostatin gene transcription by phosphorylation of CREB at serine 133. *Cell* 59 675–680. 10.1016/0092-8674(89)90013-5 2573431

[B43] Grigg J. J. Brew H. M. Tempel B. L. (2000). Differential expression of voltage-gated potassium channel genes in auditory nuclei of the mouse brainstem. *Hear. Res.* 140 77–90. 10.1016/s0378-5955(99)00187-2 10675636

[B44] Grothe B. Pecka M. (2014). The natural history of sound localization in mammals–a story of neuronal inhibition. *Front. Neural Circuits* 8:116. 10.3389/fncir.2014.00116 25324726 PMC4181121

[B45] Grothe B. Pecka M. Mcalpine D. (2010). Mechanisms of sound localization in mammals. *Physiol. Rev.* 90 983–1012. 10.1152/physrev.00026.2009 20664077

[B46] Hall S. S. Walter E. Sherman E. Hoeft F. Reiss A. L. (2009). The neural basis of auditory temporal discrimination in girls with fragile X syndrome. *J. Neurodev. Disord.* 1 91–99. 10.1007/s11689-009-9007-x 19890439 PMC2772079

[B47] Hardman R. M. Forsythe I. D. (2009). Ether-a-go-go-related gene K+ channels contribute to threshold excitability of mouse auditory brainstem neurons. *J. Physiol.* 587 2487–2497. 10.1113/jphysiol.2009.170548 19359372 PMC2714015

[B48] Haykin S. Chen Z. (2005). The cocktail party problem. *Neural Comput.* 17 1875–1902. 15992485 10.1162/0899766054322964

[B49] He K. McCord M. C. Hartnett K. A. Aizenman E. (2015). Regulation of pro-apoptotic phosphorylation of Kv2.1 K+ channels. *PLoS One* 10:e0129498. 10.1371/journal.pone.0129498 26115091 PMC4482604

[B50] Held H. (1893). Die centrale Gehörleitung. *Arch. Anat. Physiol. Anat. Abt.* 17, 201–248.

[B51] Hermann J. Pecka M. Von Gersdorff H. Grothe B. Klug A. (2007). Synaptic transmission at the calyx of Held under in vivo like activity levels. *J. Neurophysiol.* 98 807–820. 10.1152/jn.00355.2007 17507501

[B52] Holt A. G. Asako M. Duncan R. K. Lomax C. A. Juiz J. M. Altschuler R. A. (2006). Deafness associated changes in expression of two-pore domain potassium channels in the rat cochlear nucleus. *Hear. Res.* 21 146–153. 10.1016/j.heares.2006.03.009 16650703 PMC4581595

[B53] Huang C. Y. Chu D. Hwang W. C. Tsaur M. L. (2012). Coexpression of high-voltage-activated ion channels Kv3.4 and Cav1.2 in pioneer axons during pathfinding in the developing rat forebrain. *J. Comp. Neurol.* 520 3650–3672. 10.1002/cne.23119 22473424

[B54] Ikematsu N. Dallas M. L. Ross F. A. Lewis R. W. Rafferty J. N. David J. A. (2011). Phosphorylation of the voltage-gated potassium channel Kv2.1 by AMP-activated protein kinase regulates membrane excitability. *Proc. Natl. Acad. Sci. U.S.A.* 108 18132–18137. 10.1073/pnas.1106201108 22006306 PMC3207650

[B55] Ishikawa T. Nakamura Y. Saitoh N. Li W. B. Iwasaki S. Takahashi T. (2003). Distinct roles of Kv1 and Kv3 potassium channels at the calyx of Held presynaptic terminal. *J. Neurosci.* 23 10445–10453. 10.1523/jneurosci.23-32-10445.2003 14614103 PMC6741004

[B56] Johnston J. Griffin S. J. Baker C. Forsythe I. D. (2008a). Kv4 (A-type) potassium currents in the mouse medial nucleus of the trapezoid body. *Eur. J. Neurosci.* 27 1391–1399. 10.1111/j.1460-9568.2008.06116.x 18364020

[B57] Johnston J. Griffin S. J. Baker C. Skrzypiec A. Chernova T. Forsythe I. D. (2008b). Initial segment Kv2.2 channels mediate a slow delayed rectifier and maintain high frequency action potential firing in medial nucleus of the trapezoid body neurons. *J. Physiol.* 586 3493–3509. 10.1113/jphysiol.2008.153734 18511484 PMC2538803

[B58] Joris P. X. Trussell L. O. (2018). The calyx of Held: a hypothesis on the need for reliable timing in an intensity-difference encoder. *Neuron* 100 534–549. 10.1016/j.neuron.2018.10.026 30408442 PMC6263157

[B59] Joris P. X. Smith P. H. Yin T. C. (1994). Enhancement of neural synchronization in the anteroventral cochlear nucleus. II. Responses in the tuning curve tail. *J. Neurophysiol.* 71 1037–1051. 10.1152/jn.1994.71.3.1037 8201400

[B60] Kaczmarek L. K. (2006). Non-conducting functions of voltage-gated ion channels. *Nat. Rev. Neurosci.* 7 761–771. 10.1038/nrn1988 16988652

[B61] Kaczmarek L. K. (2012). Gradients and modulation of K(+) channels optimize temporal accuracy in networks of auditory neurons. *PLoS Comput. Biol.* 8:e1002424. 10.1371/journal.pcbi.1002424 22438799 PMC3305353

[B62] Kaczmarek L. K. (2013). Slack, slick and sodium-activated potassium channels. *ISRN Neurosci.* 2013:354262. 10.1155/2013/354262 PMC3850776 24319675

[B63] Kaczmarek L. K. (2019). “Extraction of auditory information by modulation of neuronal ion channels,” in *The Oxford Handbook of the Auditory Brainstem*, ed. Kandler K. (Oxford: OUP), 10.1093/oxfordhb/9780190849061.013.23

[B64] Kaczmarek L. K. (2020). “Excitable membrane properties of neurons,” in *The Oxford Handbook of Neuronal Ion Channels*, ed. Bhattacharjee A. (Oxford: OUP), 10.1093/oxfordhb/9780190669164.013.20

[B65] Kaczmarek L. K. Aldrich R. W. Chandy K. G. Grissmer S. Wei A. D. Wulff H. (2017). International union of basic and clinical pharmacology. C. Nomenclature and properties of calcium-activated and sodium-activated potassium channels. *Pharmacol. Rev.* 69 1–11. 10.1124/pr.116.012864 28267675 PMC11060434

[B66] Kaczmarek L. K. Zhang Y. (2017). Kv3 channels: enablers of rapid firing, neurotransmitter release, and neuronal endurance. *Physiol. Rev.* 97 1431–1468. 10.1152/physrev.00002.2017 28904001 PMC6151494

[B67] Kaczmarek L. K. Bhattacharjee A. Desai R. Gan L. Song P. Von Hehn C. A. (2005). Regulation of the timing of MNTB neurons by short-term and long-term modulation of potassium channels. *Hear. Res.* 206 133–145. 10.1016/j.heares.2004.11.023 16081004

[B68] Kaczmarek L. K. Wu X.-S. Subramanian S. Xia J. Zhang Y. El-Hassar L. (2019). The Kv3.3 potassium channel controls endocytosis by organizing the actin cytoskeleton at nerve terminals. *Neurosci. Abstr.* (CD ROM), 121.29.

[B69] Kanemasa T. Gan L. Perney T. M. Wang L. Y. Kaczmarek L. K. (1995). Electrophysiological and pharmacological characterization of a mammalian Shaw channel expressed in NIH 3T3 fibroblasts. *J. Neurophysiol.* 74 207–217. 10.1152/jn.1995.74.1.207 7472324

[B70] Karcz A. Hennig M. H. Robbins C. A. Tempel B. L. Rubsamen R. Kopp-Scheinpflug C. (2011). Low-voltage activated Kv1.1 subunits are crucial for the processing of sound source location in the lateral superior olive in mice. *J. Physiol.* 589 1143–1157. 10.1113/jphysiol.2010.203331 21224222 PMC3060593

[B71] Karschin C. Wischmeyer E. Preisig-Muller R. Rajan S. Derst C. Grzeschik K. H. (2001). Expression pattern in brain of TASK-1, TASK-3, and a tandem pore domain K(+) channel subunit, TASK-5, associated with the central auditory nervous system. *Mol. Cell. Neurosci.* 18 632–648. 10.1006/mcne.2001.1045 11749039

[B72] Kim G. E. Kaczmarek L. K. (2014). Emerging role of the KCNT1 Slack channel in intellectual disability. *Front. Cell. Neurosci.* 8:209. 10.3389/fncel.2014.00209 25120433 PMC4112808

[B73] Kopp-Scheinpflug C. Fuchs K. Lippe W. R. Tempel B. L. Rubsamen R. (2003a). Decreased temporal precision of auditory signaling in Kcna1-null mice: an electrophysiological study in vivo. *J. Neurosci.* 23 9199–9207. 10.1523/jneurosci.23-27-09199.2003 14534254 PMC6740830

[B74] Kopp-Scheinpflug C. Lippe W. R. Dorrscheidt G. J. Rubsamen R. (2003b). The medial nucleus of the trapezoid body in the gerbil is more than a relay: comparison of pre- and postsynaptic activity. *J. Assoc. Res. Otolaryngol.* 4 1–23. 10.1007/s10162-002-2010-5 12098017 PMC3202451

[B75] Kopp-Scheinpflug C. Tolnai S. Malmierca M. S. Rubsamen R. (2008). The medial nucleus of the trapezoid body: comparative physiology. *Neuroscience* 154 160–170. 10.1016/j.neuroscience.2008.01.088 18436383

[B76] Labro A. J. Priest M. F. Lacroix J. J. Snyders D. J. Bezanilla F. (2015). Kv3.1 uses a timely resurgent K(+) current to secure action potential repolarization. *Nat. Commun.* 6:10173. 10.1038/ncomms10173 PMC4703866 26673941

[B77] Leao K. E. Leao R. N. Deardorff A. S. Garrett A. Fyffe R. Walmsley B. (2010). Sound stimulation modulates high-threshold K(+) currents in mouse auditory brainstem neurons. *Eur. J. Neurosci.* 32 1658–1667. 10.1111/j.1460-9568.2010.07437.x 20946234 PMC3439509

[B78] Leao R. M. (2019). The ion channels and synapses responsible for the physiological diversity of mammalian lower brainstem auditory neurons. *Hear. Res.* 376 33–46. 10.1016/j.heares.2018.12.011 30606624

[B79] Leao R. N. Naves M. M. Leao K. E. Walmsley B. (2006a). Altered sodium currents in auditory neurons of congenitally deaf mice. *Eur. J. Neurosci.* 24 1137–1146. 10.1111/j.1460-9568.2006.04982.x 16930439

[B80] Leao R. N. Sun H. Svahn K. Berntson A. Youssoufian M. Paolini A. G. (2006b). Topographic organization in the auditory brainstem of juvenile mice is disrupted in congenital deafness. *J. Physiol.* 571 563–578. 10.1113/jphysiol.2005.098780 16373385 PMC1805808

[B81] Lee A. Fakler B. Kaczmarek L. K. Isom L. L. (2014). More than a pore: ion channel signaling complexes. *J. Neurosci.* 34 15159–15169. 10.1523/jneurosci.3275-14.2014 25392484 PMC4228125

[B82] Li W. Kaczmarek L. K. Perney T. M. (2001). Localization of two high-threshold potassium channel subunits in the rat central auditory system. *J. Comp. Neurol.* 437 196–218. 10.1002/cne.1279 11494252

[B83] Liu S. J. Kaczmarek L. K. (1998). The expression of two splice variants of the Kv3.1 potassium channel gene is regulated by different signaling pathways. *J. Neurosci.* 18 2881–2890. 10.1523/jneurosci.18-08-02881.1998 9526005 PMC6792597

[B84] Liu S. Q. Kaczmarek L. K. (1998). Depolarization selectively increases the expression of the Kv3.1 potassium channel in developing inferior colliculus neurons. *J. Neurosci.* 18 8758–8769. 10.1523/jneurosci.18-21-08758.1998 9786983 PMC6793528

[B85] Lorteije J. A. Rusu S. I. Kushmerick C. Borst J. G. (2009). Reliability and precision of the mouse calyx of Held synapse. *J. Neurosci.* 29 13770–13784. 10.1523/jneurosci.3285-09.2009 19889989 PMC6666705

[B86] Luneau C. J. Williams J. B. Marshall J. Levitan E. S. Oliva C. Smith J. S. (1991). Alternative splicing contributes to K+ channel diversity in the mammalian central nervous system. *Proc. Natl. Acad. Sci. U.S.A.* 88 3932–3936. 10.1073/pnas.88.9.3932 2023941 PMC51567

[B87] Macica C. M. Kaczmarek L. K. (2001). Casein kinase 2 determines the voltage dependence of the Kv3.1 channel in auditory neurons and transfected cells. *J. Neurosci.* 21 1160–1168. 10.1523/jneurosci.21-04-01160.2001 11160386 PMC6762230

[B88] Macica C. M. Von Hehn C. A. Wang L. Y. Ho C. S. Yokoyama S. Joho R. H. (2003). Modulation of the kv3.1b potassium channel isoform adjusts the fidelity of the firing pattern of auditory neurons. *J. Neurosci.* 23 1133–1141. 10.1523/jneurosci.23-04-01133.2003 12598601 PMC6742259

[B89] Manis P. B. Marx S. O. (1991). Outward currents in isolated ventral cochlear nucleus neurons. *J. Neurosci.* 11 2865–2880. 10.1523/jneurosci.11-09-02865.1991 1880553 PMC6575259

[B90] Mathews P. J. Jercog P. E. Rinzel J. Scott L. L. Golding N. L. (2010). Control of submillisecond synaptic timing in binaural coincidence detectors by K(v)1 channels. *Nat. Neurosci.* 13 601–609. 10.1038/nn.2530 20364143 PMC3375691

[B91] McCullagh E. A. Rotschafer S. E. Auerbach B. D. Klug A. Kaczmarek L. K. Cramer K. S. (2020). Mechanisms underlying auditory processing deficits in Fragile X syndrome. *FASEB J.* 34 3501–3518. 10.1096/fj.201902435r 32039504 PMC7347277

[B92] McDermott J. H. (2009). The cocktail party problem. *Curr. Biol.* 19 R1024–R1027. 19948136 10.1016/j.cub.2009.09.005

[B93] Middlebrooks J. C. Nick H. S. Subramony S. H. Advincula J. Rosales R. L. Lee L. V. (2013). Mutation in the Kv3.3 voltage-gated potassium channel causing spinocerebellar ataxia 13 disrupts sound-localization mechanisms. *PLoS One* 8:e76749. 10.1371/journal.pone.0076749 24116147 PMC3792041

[B94] Moore M. J. Caspary D. M. (1983). Strychnine blocks binaural inhibition in lateral superior olivary neurons. *J. Neurosci.* 3 237–242. 10.1523/jneurosci.03-01-00237.1983 6822858 PMC6564582

[B95] Münte T. F. Kohlmetz C. Nager W. Altenmuller E. (2001). Superior auditory spatial tuning in conductors. *Nature* 409:580. 10.1038/35054668 11214309

[B96] Nabel A. L. Callan A. R. Gleiss S. A. Kladisios N. Leibold C. Felmy F. (2019). Distinct distribution patterns of potassium channel sub-units in somato-dendritic compartments of neurons of the medial superior olive. *Front. Cell. Neurosci.* 13:38. 10.3389/fncel.2019.00038 30837841 PMC6390502

[B97] Nager W. Kohlmetz C. Altenmuller E. Rodriguez-Fornells A. Münte T. F. (2003). The fate of sounds in conductors’ brains: an ERP study. *Brain Res. Cogn. Brain Res.* 17 83–93. 10.1016/s0926-6410(03)00083-1 12763195

[B98] Niemeyer M. I. Cid L. P. Gonzalez W. Sepulveda F. V. (2016). Gating, regulation, and structure in K2P K+ channels: in varietate concordia? *Mol. Pharmacol.* 90 309–317. 10.1124/mol.116.103895 27268784

[B99] Oertel D. (1983). Synaptic responses and electrical properties of cells in brain slices of the mouse anteroventral cochlear nucleus. *J. Neurosci.* 3 2043–2053. 10.1523/jneurosci.03-10-02043.1983 6619923 PMC6564561

[B100] Oertel D. Shatadal S. Cao X. J. (2008). In the ventral cochlear nucleus Kv1.1 and subunits of HCN1 are colocalized at surfaces of neurons that have low-voltage-activated and hyperpolarization-activated conductances. *Neuroscience* 154 77–86. 10.1016/j.neuroscience.2008.01.085 18424000 PMC2493296

[B101] Perney T. M. Kaczmarek L. K. (1997). Localization of a high threshold potassium channel in the rat cochlear nucleus. *J. Comp. Neurol.* 386 178–202. 10.1002/(sici)1096-9861(19970922)386:2<178::aid-cne2>3.0.co;2-z 9295146

[B102] Perney T. M. Marshall J. Martin K. A. Hockfield S. Kaczmarek L. K. (1992). Expression of the mRNAs for the Kv3.1 potassium channel gene in the adult and developing rat brain. *J. Neurophysiol.* 68 756–766. 10.1152/jn.1992.68.3.756 1432046

[B103] Rasmussen H. B. Trimmer J. S. (2019). “The voltage-dependent K+ channel family,” in *The Oxford Handbook of Neuronal Ion Channels*, ed. Bhattacharjee A. (Oxford: OUP), 10.1093/oxfordhb/9780190669164.013.1

[B104] Remme M. W. Donato R. Mikiel-Hunter J. Ballestero J. A. Foster S. Rinzel J. (2014). Subthreshold resonance properties contribute to the efficient coding of auditory spatial cues. *Proc. Natl. Acad. Sci. U.S.A.* 111 E2339–E2348. 24843153 10.1073/pnas.1316216111 PMC4050603

[B105] Richter J. D. Bassell G. J. Klann E. (2015). Dysregulation and restoration of translational homeostasis in fragile X syndrome. *Nat. Rev. Neurosci.* 16 595–605. 10.1038/nrn4001 26350240 PMC4688896

[B106] Robbins C. A. Tempel B. L. (2012). Kv1.1 and Kv1.2: similar channels, different seizure models. *Epilepsia* 53(Suppl. 1) 134–141. 10.1111/j.1528-1167.2012.03484.x 22612818

[B107] Roberts J. Hennon E. A. Anderson K. Roush J. Gravel J. Skinner M. (2005). Auditory brainstem responses in young males with Fragile X syndrome. *J. Speech Lang. Hear. Res.* 48 494–500. 10.1044/1092-4388(2005/034) 15989407

[B108] Rojas D. C. Benkers T. L. Rogers S. J. Teale P. D. Reite M. L. Hagerman R. J. (2001). Auditory evoked magnetic fields in adults with fragile X syndrome. *Neuroreport* 12 2573–2576. 10.1097/00001756-200108080-00056 11496151

[B109] Rothman J. S. Manis P. B. (2003). The roles potassium currents play in regulating the electrical activity of ventral cochlear nucleus neurons. *J. Neurophysiol.* 89 3097–3113. 10.1152/jn.00127.2002 12783953

[B110] Rotschafer S. E. Razak K. A. (2014). Auditory processing in fragile x syndrome. *Front. Cell. Neurosci.* 8:19. 10.3389/fncel.2014.00019 24550778 PMC3912505

[B111] Rusznak Z. Bakondi G. Pocsai K. Por A. Kosztka L. Pal B. (2008). Voltage-gated potassium channel (Kv) subunits expressed in the rat cochlear nucleus. *J. Histochem. Cytochem.* 56 443–465. 10.1369/jhc.2008.950303 18256021 PMC2324191

[B112] Saher M. H. (2020). *Investigation of The Physiological Role Of The Electrically Silent K2P Subunit Task5 in The Auditory Brainstem.* Ph.D. thesis, University of Heidelberg, Germany.

[B113] Santi C. M. Ferreira G. Yang B. Gazula V. R. Butler A. Wei A. (2006). Opposite regulation of Slick and Slack K+ channels by neuromodulators. *J. Neurosci.* 26 5059–5068. 10.1523/jneurosci.3372-05.2006 16687497 PMC6674240

[B114] Schneggenburger R. Forsythe I. D. (2006). The calyx of Held. *Cell Tissue Res.* 326 311–337. 16896951 10.1007/s00441-006-0272-7

[B115] Schwarz D. W. Puil E. (1997). Firing properties of spherical bushy cells in the anteroventral cochlear nucleus of the gerbil. *Hear. Res.* 114 127–138. 10.1016/s0378-5955(97)00162-7 9447927

[B116] Scott L. L. Mathews P. J. Golding N. L. (2010). Perisomatic voltage-gated sodium channels actively maintain linear synaptic integration in principal neurons of the medial superior olive. *J. Neurosci.* 30 2039–2050. 10.1523/jneurosci.2385-09.2010 20147532 PMC2827923

[B117] Singer-Lahat D. Dascal N. Lotan I. (1999). Modal behavior of the Kv1.1 channel conferred by the Kvbeta1.1 subunit and its regulation by dephosphorylation of Kv1.1. *Pflugers Arch.* 439 18–26. 10.1007/s004240051123 10650996

[B118] Smith P. H. Rhode W. S. (1987). Characterization of HRP-labeled globular bushy cells in the cat anteroventral cochlear nucleus. *J. Comp. Neurol.* 266 360–375. 10.1002/cne.902660305 3693616

[B119] Song P. Kaczmarek L. K. (2006). Modulation of Kv3.1b potassium channel phosphorylation in auditory neurons by conventional and novel protein kinase C isozymes. *J. Biol. Chem.* 281 15582–15591. 10.1074/jbc.m512866200 16595659

[B120] Song P. Yang Y. Barnes-Davies M. Bhattacharjee A. Hamann M. Forsythe I. D. (2005). Acoustic environment determines phosphorylation state of the Kv3.1 potassium channel in auditory neurons. *Nat. Neurosci.* 8 1335–1342. 10.1038/nn1533 16136041

[B121] St Clair D. M. Blackwood D. H. Oliver C. J. Dickens P. (1987). P3 abnormality in fragile X syndrome. *Biol. Psychiatry* 22 303–312. 10.1016/0006-3223(87)90148-x 2949781

[B122] Steinert J. R. Kopp-Scheinpflug C. Baker C. Challiss R. A. J. Mistry R. Haustein M. D. (2008). Nitric oxide is a volume transmitter regulating postsynaptic excitability at a glutamatergic synapse. *Neuron* 60 642–656. 19038221 10.1016/j.neuron.2008.08.025

[B123] Steinert J. R. Robinson S. W. Tong H. Haustein M. D. Kopp-Scheinpflug C. Forsythe I. D. (2011). Nitric oxide is an activity-dependent regulator of target neuron intrinsic excitability. *Neuron* 71 291–305. 21791288 10.1016/j.neuron.2011.05.037 PMC3245892

[B124] Strumbos J. G. Brown M. R. Kronengold J. Polley D. B. Kaczmarek L. K. (2010a). Fragile X mental retardation protein is required for rapid experience-dependent regulation of the potassium channel Kv3.1b. *J. Neurosci.* 30 10263–10271. 20685971 10.1523/JNEUROSCI.1125-10.2010 PMC3485078

[B125] Strumbos J. G. Polley D. B. Kaczmarek L. K. (2010b). Specific and rapid effects of acoustic stimulation on the tonotopic distribution of Kv3.1b potassium channels in the adult rat. *Neuroscience* 167 567–572. 20219640 10.1016/j.neuroscience.2010.02.046 PMC2854512

[B126] Svirskis G. Kotak V. Sanes D. H. Rinzel J. (2004). Sodium along with low-threshold potassium currents enhance coincidence detection of subthreshold noisy signals in MSO neurons. *J. Neurophysiol.* 91 2465–2473. 14749317 10.1152/jn.00717.2003 PMC3683536

[B127] Taschenberger H. von Gersdorff H. (2000). Fine-tuning an auditory synapse for speed and fidelity: developmental changes in presynaptic waveform, EPSC kinetics, and synaptic plasticity. *J. Neurosci.* 20 9162–9173. 10.1523/JNEUROSCI.20-24-09162.2000 11124994 PMC6773022

[B128] Taskin B. Von Schoubye N. L. Sheykhzade M. Bastlund J. F. Grunnet M. Jespersen T. (2015). Biophysical characterization of KV3.1 potassium channel activating compounds. *Eur. J. Pharmacol.* 758 164–170. 10.1016/j.ejphar.2015.03.061 25845309

[B129] Tollin D. J. (2003). The lateral superior olive: a functional role in sound source localization. *Neuroscientist* 9 127–143. 10.1177/1073858403252228 12708617

[B130] Tollin D. J. (2009). “Development of sound localization,” in *Oxford Handbook of Developmental Behavioral Neuroscience*, eds Blumberg M. S. Freeman J. H. Robinson S. R. (Oxford: OUP)

[B131] Tong H. Kopp-Scheinpflug C. Pilati N. Robinson S. W. Sinclair J. L. Steinert J. R. (2013). Protection from noise-induced hearing loss by Kv2.2 potassium currents in the central medial olivocochlear system. *J. Neurosci.* 33 9113–9121. 10.1523/JNEUROSCI.5043-12.2013 23699522 PMC5503134

[B132] Tong H. Steinert J. R. Robinson S. W. Chernova T. Read D. J. Oliver D. L. (2010). Regulation of Kv channel expression and neuronal excitability in rat medial nucleus of the trapezoid body maintained in organotypic culture. *J. Physiol.* 588 1451–1468. 10.1113/jphysiol.2009.186676 20211981 PMC2876802

[B133] Vacher H. Trimmer J. S. (2011). Diverse roles for auxiliary subunits in phosphorylation-dependent regulation of mammalian brain voltage-gated potassium channels. *Pflugers Arch.* 462 631–643. 10.1007/s00424-011-1004-8 21822597 PMC3371647

[B134] Van der Molen M. J. Van Der Molen M. W. Ridderinkhof K. R. Hamel B. C. Curfs L. M. Ramakers G. J. (2012). Auditory and visual cortical activity during selective attention in fragile X syndrome: a cascade of processing deficiencies. *Clin. Neurophysiol.* 123 720–729. 10.1016/j.clinph.2011.08.023 21958658

[B135] von Hehn C. A. Bhattacharjee A. Kaczmarek L. K. (2004). Loss of Kv3.1 tonotopicity and alterations in cAMP response element-binding protein signaling in central auditory neurons of hearing impaired mice. *J. Neurosci.* 24 1936–1940. 10.1523/JNEUROSCI.4554-03.2004 14985434 PMC6730406

[B136] Wang L. Y. Kaczmarek L. K. (1998). High-frequency firing helps replenish the readily releasable pool of synaptic vesicles. *Nature* 394 384–388. 10.1038/28645 9690475

[B137] Wang L. Y. Gan L. Forsythe I. D. Kaczmarek L. K. (1998). Contribution of the Kv3.1 potassium channel to high-frequency firing in mouse auditory neurones. *J. Physiol.* 509(Pt 1) 183–194. 10.1111/j.1469-7793.1998.183bo.x 9547392 PMC2230948

[B138] Winklhofer M. Matthias K. Seifert G. Stocker M. Sewing S. Herget T. (2003). Analysis of phosphorylation-dependent modulation of Kv1.1 potassium channels. *Neuropharmacology* 44 829–842. 10.1016/S0028-3908(03)00070-4 12681381

[B139] Yang B. Desai R. Kaczmarek L. K. (2007a). Slack and Slick K(Na) channels regulate the accuracy of timing of auditory neurons. *J. Neurosci.* 27 2617–2627. 10.1523/JNEUROSCI.5308-06.2007 17344399 PMC6672517

[B140] Yang J. W. Vacher H. Park K. S. Clark E. Trimmer J. S. (2007b). Trafficking-dependent phosphorylation of Kv1.2 regulates voltage-gated potassium channel cell surface expression. *Proc. Natl. Acad. Sci. U.S.A.* 104 20055–20060. 10.1073/pnas.0708574104 18056633 PMC2148421

[B141] Yang Y. M. Arsenault J. Bah A. Krzeminski M. Fekete A. Chao O. Y. (2018). Identification of a molecular locus for normalizing dysregulated GABA release from interneurons in the Fragile X brain. *Mol. Psychiatry* 25 2017–2035. 10.1038/s41380-018-0240-0 30224722 PMC7473840

[B142] Yin T. C. T. Smith P. H. Joris P. X. (2019). Neural mechanisms of binaural processing in the auditory brainstem. *Compr. Physiol.* 9 1503–1575. 10.1002/cphy.c180036 31688966

[B143] Zhang Y. Kaczmarek L. K. (2016). Kv3.3 potassium channels and spinocerebellar ataxia. *J. Physiol.* 594 4677–4684. 10.1113/JP271343 26442672 PMC4983625

[B144] Zhang Y. Brown M. R. Hyland C. Chen Y. Kronengold J. Fleming M. R. (2012). Regulation of neuronal excitability by interaction of fragile X mental retardation protein with slack potassium channels. *J. Neurosci.* 32 15318–15327. 10.1523/JNEUROSCI.2162-12.2012 23115170 PMC3518385

[B145] Zhang Y. Zhang X. F. Fleming M. R. Amiri A. El-Hassar L. Surguchev A. A. (2016). Kv3.3 channels bind Hax-1 and Arp2/3 to assemble a stable local actin network that regulates channel gating. *Cell* 165 434–448. 10.1016/j.cell.2016.02.009 26997484 PMC4826296

[B146] Zion Golumbic E. M. Ding N. Bickel S. Lakatos P. Schevon C. A. Mckhann G. M. (2013). Mechanisms underlying selective neuronal tracking of attended speech at a “cocktail party”. *Neuron* 77 980–991. 10.1016/j.neuron.2012.12.037 23473326 PMC3891478

[B147] Zuniga L. Zuniga R. (2016). Understanding the Cap Structure in K2P Channels. *Front. Physiol.* 7:228. 10.3389/fphys.2016.00228 27378938 PMC4906011

